# The ABC transporters in *Candidatus* Liberibacter asiaticus

**DOI:** 10.1002/prot.24147

**Published:** 2012-07-31

**Authors:** Wenlin Li, Qian Cong, Jimin Pei, Lisa N Kinch, Nick V Grishin

**Affiliations:** 1 Department of Biochemistry and Department of Biophysics, University of Texas Southwestern Medical CenterDallas, Texas 75390-9050; 2 Howard Hughes Medical Institute, University of Texas Southwestern Medical CenterDallas, Texas 75390-9050

**Keywords:** genomic annotation, function prediction, ATPase, transmembrane protein, multiple sequence alignment, phylogenetic tree, protein homology, structure comparison

## Abstract

*Candidatus* Liberibacter asiaticus (*Ca.* L. asiaticus) is a Gram-negative bacterium and the pathogen of Citrus Greening disease (Huanglongbing, HLB). As a parasitic bacterium, *Ca.* L. asiaticus harbors ABC transporters that play important roles in exchanging chemical compounds between *Ca.* L. asiaticus and its host. Here, we analyzed all the ABC transporter-related proteins in *Ca.* L. asiaticus. We identified 14 ABC transporter systems and predicted their structures and substrate specificities. In-depth sequence and structure analysis including multiple sequence alignment, phylogenetic tree reconstruction, and structure comparison further support their function predictions. Our study shows that this bacterium could use these ABC transporters to import metabolites (amino acids and phosphates) and enzyme cofactors (choline, thiamine, iron, manganese, and zinc), resist to organic solvent, heavy metal, and lipid-like drugs, maintain the composition of the outer membrane (OM), and secrete virulence factors. Although the features of most ABC systems could be deduced from the abundant experimental data on their orthologs, we reported several novel observations within ABC system proteins. Moreover, we identified seven nontransport ABC systems that are likely involved in virulence gene expression regulation, transposon excision regulation, and DNA repair. Our analysis reveals several candidates for further studies to understand and control the disease, including the type I virulence factor secretion system and its substrate that are likely related to *Ca.* L. asiaticus pathogenicity and the ABC transporter systems responsible for bacterial OM biosynthesis that are good drug targets.

## INTRODUCTION

Citrus Greening, also known as Huanglongbing (HLB), is one of the most destructive diseases of citrus. It was first reported in the early 20th century^1^,^2^ and has developed into a major threat for the citrus industry in China, Brazil, and the Eastern United States.^3^,^4^ The symptoms of HLB mainly include yellow shoots, chlorosis leaves, premature defoliation, and aborted fruits, followed by the eventual death of the entire plant.^5^,^6^ The causal agents of HLB are believed to be three closely related bacteria in the *Candidatus* Liberibacter (*Ca.* L.) genus, that is, *Ca.* L. asiaticus, *Ca*. L. americanus, and *Ca*. L. africanus.^5^ Among them, *Ca.* L. asiaticus is the most widespread and thus attracts the most attention from researchers.^7^

*Ca.* L. asiaticus is a Gram-negative alphaproteobacterium. Phylogenetic studies, using 16S rRNA and other genes,^8^,^9^ placed this bacterium in the family of *Rhizobiaceae*. The long branch of *Ca.* L. asiaticus in the phylogenetic tree reveals rapid evolution of this pathogen.^8^ The bacterium is transmitted among citrus plants by the piercing-sucking insects, citrus psyllids (*Diaphorina citri* Kuwayama and *Trioza erytreae*). In the plant, *Ca.* L. asiaticus resides mainly in the phloem tissue.^5^,^6^ Efforts have been made to understand the mechanism of the disease.^10^–^12^ However, difficulty in maintaining the bacterium in culture makes it challenging to carry out any experiments directly on *Ca.* L. asiaticus. Recently, the complete genome sequence^8^ of the bacterium was obtained, which opened the possibility of getting insight into the pathogen and the disease by careful analysis of the genome with computational methods.

Here, we focus on the ATP-binding cassette (ABC) systems of the bacterium. ABC systems function in several central cellular processes such as nutrient uptake, drug export, and gene regulation.^13^ Based on current phylogenetic analysis, the ABC systems can be divided into three classes: exporters, nontransporting ABC proteins, and a third class that is mostly composed of importers.^14^,^15^ The essential ABC system component is an ABC-type ATPase (also named ABC protein or Nucleotide Binding Domain, NBD). The ABC-type ATPase contains a series of highly conserved sequence motifs, including Walker A and Walker B, which are common for all P-loop NTPases,^16^ and Walker C, the signature of the ABC-type ATPase.^17^ Walker A and Walker B are crucial for binding and hydrolyzing ATP. As ABC-type ATPases mostly function as homodimers, Walker C is responsible for binding ATP on the side opposite to Walker A and Walker B and is essential for cross-talks between the two monomers.

Most ABC systems include transmembrane proteins and function as transporters. One ABC transporter consists of at least four domains, that is, two transmembrane domains (TMDs) and two NBDs. Pseudo-centrosymmetric dimers formed by two homologous TMDs with similar structures are prevalent. The only known asymmetrically dimeric ABC transporters, the ECF transporters,^18^,^19^ have two possibly structurally different TMDs, a T-component TMD and an S-component TMD. The TMDs of most ABC-type transporters fall into four clans in the Protein families database (Pfam)^20^: “ABC transporter membrane domain” clan (CL0241), “ABC-2-transporter-like” clan (CL0181), “membrane and transport protein” clan (CL0142), and “BPD transporter like” clan (CL0404). Several known structures suggest that the TMD should dock into the NBD by a “coupling helix”,^21^ which coordinates a conformational change caused by ATP-hydrolysis. A number of ABC systems also include periplasmic components that are responsible for transporting the substrates across the periplasmic space. Periplasmic-binding proteins (PBPs) are used by many importers to recognize substrates and initialize the transporting cycle by interacting with the TMD.^22^ Similarly, some exporters, especially those from Gram-negative bacteria, use a series of auxiliary proteins [periplasmic proteins and/or outer membrane (OM) proteins] to route the cargo across the periplasmic space. Here, we refer to the PBPs, auxiliary proteins, TMD-containing proteins, and NBD-containing proteins in the following text as “ABC system proteins.”

In addition to primary active transport, ABC transporter activity is thought to be related to virulence in some Gram-negative bacteria.^23^–^26^ In plants infected by *Ca.* L. asiaticus, the ABC transporters may contribute to host metabolic imbalances and thus the Citrus Greening disease symptoms.^8^ Given the important roles of ABC transporters and their possible involvement in pathogenicity, analysis of these ABC transporters will help us to understand the metabolism of the bacterium and the mechanism of the disease. In this article, we report a detailed study of all ABC transporters in the *Ca.* L. asiaticus. We collected all potential ABC system proteins in the proteome and identified 14 ABC transporter systems and 7 nontransporting ABC proteins. Combining different computational methods, we predicted the structure and substrate specificity of each ABC transporter.

## MATERIALS AND METHODS

### Identification of ABC system proteins

*Ca.* L. asiaticus protein sequences were downloaded from the NCBI Genbank database (ftp://ftp.ncbi.nih.gov/genbank/genomes/Bacteria/Candidatus_Liberibacter_asiaticus_psy62_uid29835), and additional proteins predicted by the SEED^27^ (http://pseed.theseed.org/seedviewer.cgi), but missed by NCBI, were added. The relevant information and annotations of these proteins from NCBI (http://www.ncbi.nlm.nih.gov/nuccore/CP001677), Cluster of Orthologous Groups (COGs),^28^ and the SEED were taken as references. The protein annotations by NCBI, COG and the SEED were examined manually to obtain a primary list of ABC system proteins, followed by two additional approaches to ensure a complete list. First, starting from the proteins in the primary list and the sequence profiles of known ABC system protein families in Pfam, we used PSI-BLAST^29^,^30^ and HHsearch^31^ to identify homologous proteins from the *Ca.* L. asiaticus proteome. Second, assisted by our comprehensive database of the *Ca.* L. asiaticus proteome (http://prodata.swmed.edu/liberibacter_asiaticus/), we manually curated all the proteins to ensure that all ABC system proteins were included in our list.

### Substrate specificity and structure prediction of assembled ABC systems

For each predicted *Ca.* L. asiaticus ABC system protein, we applied PSI-BLAST, RPS-BLAST,^32^ and HHsearch to detect homologous proteins, protein families, and conserved domains,^33^ paying special attention to close homologs with experimentally verified functions. Sequence comparison between *Ca.* L. asiaticus and these homologs assisted by the sequence clustering tool CLANS^34^ served as the primary evidence for our function assignments. Based on these assignments, the genomic context of each protein retrieved from the SEED, and the functional association networks between proteins detected by STRING,^35^ we assembled these protein components into ABC transport complexes. The closest homologous proteins with 3D crystal structures judged by confidence, coverage, and match of conserved residues were manually selected as structure templates. MODELLER^36^ was then applied to these templates to obtain a structure model for each protein. For potential TMDs and PBPs, the transmembrane helices and signal peptides were detected by TMHMM,^37^ TOPPRED,^38^ HMMTOP,^39^ MEMSAT,^40^ MEMSAT_SVM,^41^ Phobius,^42^ and SignalP^43^ to reveal their topologies and confirm their subcellular localization.

### Multiple sequence alignment and phylogenetic tree of NBDs in *Ca.* L. asiaticus

We generated a multiple sequence alignment (MSA) for all the NBDs in the *Ca.* L. asiaticus proteome together with five representative protein structure templates by PROfile Multiple Alignment with predicted Local Structures and 3D constraints (PROMALS3D),^44^ followed by manual adjustments. The sequences of well-characterized ABC-type ATPases from other organisms were then added to these predicted NBD sequences to generate a common MSA. For phylogenetic reconstruction, positions with gap fraction over 10% were discarded. Phylogenetic estimation using Maximum Likelihood (PhyML) program^45^ was used to build an evolutionary tree with LG model^46^ for the substitution model, four discrete rate categories for the rate heterogeneity among sites, Nearest Neighbor Interchange for the tree improvement, and SH-like approximate Likelihood-Ratio Test^47^ for estimating the branch support.

### Sequence and structure analysis of the TMDs

We performed structure comparison on the TMDs to classify them manually on the basis of their topology and architecture. We further used PROMALS3D to construct a MSA of TMD sequences in each group, together with homologous structure templates and representative sequences that share more than 30% sequence identity with *Ca.* L. asiaticus proteins. The MSAs were then improved by manual adjustment considering both sequence patterns and structure features.

## RESULTS AND DISCUSSION

### Detection and annotation of ABC-transporters in *Ca.* L. asiaticus

A total of 55 ABC system proteins were detected in the whole genome. We identified 14 complete ABC transport systems consisting of 42 ABC-system proteins and 7 ABC-type ATPases that are likely involved in cellular processes other than transport (Table I). The remaining six potential ABC transporter components do not have confident NBD partners in the proteome, and we thus name them “orphan” ABC components (Supporting Information Table SI).

**Table I:** ABC Systems in *Ca. L. asiaticus*

NBD							TMD		Other	
			Identified ortholog^b^							
Class^a^	Family^a^	Gene locus	Name	Species^c^	e-Value	Identity^d^ (%)	Gene locus	Pfam family (clan)	Gene locus (type^e^)	Function prediction or substrate specificity
III(i)	PAO	CLIBASIA_00280	AapP	*R*. *leguminosarum*	1e^−107^	68	CLIBASIA_00275, CLIBASIA_00270	PF00528 (CL0404)	CLIBASIA_00265 (PP)	General l-amino acid
III(i)	MOI	CLIBASIA_02955	PstB	*E. coli*	7e^−75^	56	CLIBASIA_02960, CLIBASIA_02965	PF00528 (CL0404)	CLIBASIA_02970 (PP)	Phosphate
III(i)	MOI	CLIBASIA_02230	ThiQ	*S. enterica*	8e^−68^	52	CLIBASIA_02235	PF00528 (CL0404)	CLIBASIA_02240 (PP)	Thiamine (vitamin B_1_)
III(i)	OTCN	CLIBASIA_01125	ChoV	*S. meliloti*	1e^−114^	60	CLIBASIA_01130	PF00528 (CL0404)	CLIBASIA_01135 (PP)	Choline (vitamin B_p_)
III(i)	OTCN	CLIBASIA_02415	NrtD	*S. elongatus*	5e^−42^	39	CLIBASIA_02420	PF00528 (CL0404)	N/A	Possible oxoacid ion
			SsuB	*B. subtilis*	4e^−41^	38				
			TauB	*E. coli*	3e^−39^	42				
III(i)	MET	CLIBASIA_03025	ZnuC	*E. coli*	3e^−59^	45	CLIBASIA_03030	PF00950 (CL0142)	CLIBASIA_03020 (PP)	Zinc
III(i)	MET	CLIBASIA_02125	SitB	*S. typhimurium*	6e^−71^	52	CLIBASIA_02130, CLIBASIA_02135	PF00950 (CL0142)	CLIBASIA_02120 (PP)	Manganese and iron
III(i)	MKL	CLIBASIA_00090	LinL	*S. japonicum*	6e^−62^	49	CLIBASIA_00085	PF02405(N/A)	CLIBASIA_00095 (PP), CLIBASIA_00100 (PP)	Membrane lipid
III(e)	o228	CLIBASIA_03840	LolD	*E. coli*	9e^−45^	43	peg.788, peg.789^f^	PF02687 (CL0404)	CLIBASIA_03445 (PP)	Lipoprotein
III(e)	LPT	CLIBASIA_03155	LptB	*E. coli*	1e^−60^	51	CLIBASIA_01390, CLIBASIA_01395	PF03739 (CL0404)	CLIBASIA_03160 (PP), CLIBASIA_03165 (PP), CLIBASIA_01400 (OM)	Lipopolysaccharide
I	LIP	CLIBASIA_04080	MsbA	*E. coli*	5e^−51^	48	CLIBASIA_04080	PF00664 (CL0241)	N/A	Multidrug/lipid
I	LIP	CLIBASIA_00390	MsbA	*E. coli*	7e^−52^	45	CLIBASIA_00390	PF00664 (CL0241)	N/A	Multidrug/lipid
I	HMT	CLIBASIA_02315	AtmA	*C. metallidurans*	5e^−74^	54	CLIBASIA_02315	PF00664 (CL0241)	N/A	Heavy metal
I	PRT	CLIBASIA_01350	PrtD	*S. meliloti*	9e^−49^	26	CLIBASIA_01350	PF00664 (CL0241)	CLIBASIA_01355 (PP), CLIBASIA_04145 (OM)	Type I protein secretion
III(s)	ISB	CLIBASIA_04810	SufC	*E. coli*	5e^−72^	56	N/A	N/A	N/A	Fe-S assembly
II	ART	CLIBASIA_00790	ChvD	*A. tumefaciens*	0	77	N/A	N/A	N/A	Virulence gene regulation
II	ART	CLIBASIA_05125	Uup	*E. coli*	6e^−90^	35	N/A	N/A	N/A	Transposon excision regulation
II	UVR	CLIBASIA_00335	UvrA	*S. meliloti*	0	67	N/A	N/A	N/A	DNA repair
New		CLIBASIA_03185	MutS	*R. etli*	0	58	N/A	N/A	N/A	DNA repair
New		CLIBASIA_03285	RecF	*R. etli*	3e^−107^	49	N/A	N/A	N/A	DNA repair
New		CLIBASIA_05400	RecN	*E. coli*	1e^−71^	31	N/A	N/A	N/A	DNA repair

a Classes and families are defined by existing NBD family classification.^15^ In class III, (i), (e), and (s) mean importers, exporters, and soluble proteins, respectively.

b Closest BLAST hit with experimentally determined function.

c Abbreviations for species: *R*. *leguminosarum, Rhizobium leguminosarum; E. coli, Escherichia coli; S. enterica, Salmonella enterica; S. meliloti, Sinorhizobium meliloti; S. elongates*, *Synechococcus elongatus*; *B. subtilis, Bacillus subtilis; S. typhimurium, Salmonella typhimurium; S. japonicum, Sphingobium japonicum; C. metallidurans, Cupriavidus metallidurans; A. tumefaciens, Agrobacterium tumefaciens; R. etli, Rhizobium etli*.

d Sequence identity calculated according to *Ca. L. asiaticus* NBD for the transporters and *Ca. L. asiaticus* full-length protein for nontransport ATPases.

e Auxiliary/periplasmic components according to the cellular localization. PP, periplasmic protein (PBP or other periplasmic auxiliary proteins); OM, outer membrane protein.

f fProteins detected by the SEED but missed in NCBI database. They are encoded by neighboring genes in the genome and likely to function together.

### Evidence for annotations

To ensure functional annotations deduced by homologous proteins, we identified close homologs with experimentally verified function for each proposed ABC transporter NBD. Their close relationships are reinforced by reciprocal best hits detected by BLAST. Most NBDs of *Ca.* L. asiaticus share more than 40% sequence identity (shown in Table I) with their close homologs (proposed orthologs) with experimentally verified function, meeting the suggested threshold for precise function annotation transfer.^48^ Each *Ca.* L. asiaticus NBD clustered into a group (e-value cutoff: 1e^−40^) with its proposed ortholog as revealed by clustering on the basis of all-to-all BLAST sequence comparison (Supporting Information Fig. S1), with an exception of the type I secretion system (discussed below). Four NBDs, that is, CLIBASIA_02415 (Nrt/Ssu/Tau-like system NBD), CLIBASIA_0135 (type I secretion system NBD), CLIBASIA_05125 (Uup nontransport system), and CLIBASIA_05400 (RecN nontransport system) are more variable and show marginal or low-sequence identity (<40%) to their proposed orthologs. We will discuss their predictions in the functional detail section.

### Novel predictions of ABC system proteins

The original annotations of these ABC-system proteins from NCBI, SEED, COG, and KEGG (Supporting Information Table SII) were able to place them as ABC-transporter components. However, clear predictions on their substrate specificity and polarity of the transporters were absent in many cases. Although for 86% of all proteins, the most specific annotation carefully chosen from all these databases could successfully indicate the same substrate or function predictions as ours, our manual study provided or modified the annotation for seven ABC system proteins from four ABC systems including choline/acetylcholine importer (Cho system), possible oxoacid ion importer (Nrt/Ssu/Tau-like system), lipoprotein exporter (Lol system), and Uup nontransport ABC protein. The systems revised with new annotations are described later.

The TMD of lipoprotein exporter (Lol system) was absent in current NCBI and KEGG databases, possibly due to the fact that this TMD consists of two open reading frames that were considered as pseudogenes by the NCBI gene prediction pipeline. The gene prediction pipeline of the SEED, in contrast, detected these two protein fragments but failed to predict the function of the second half. Because of the presence of the intact Lol system ATPase and other essential components in *Ca.* L. asiaticus, it is unlikely that the TMD of Lol system has lost its function. Instead, in the absence of any potential sequencing error, the two protein halves may interact with each other after translation, or some type of translational frame shift mechanism may allow the successful expression of the full protein.

### Sequence and structural analysis of the NBDs in *Ca.* L. asiaticus

**Figure 1:**
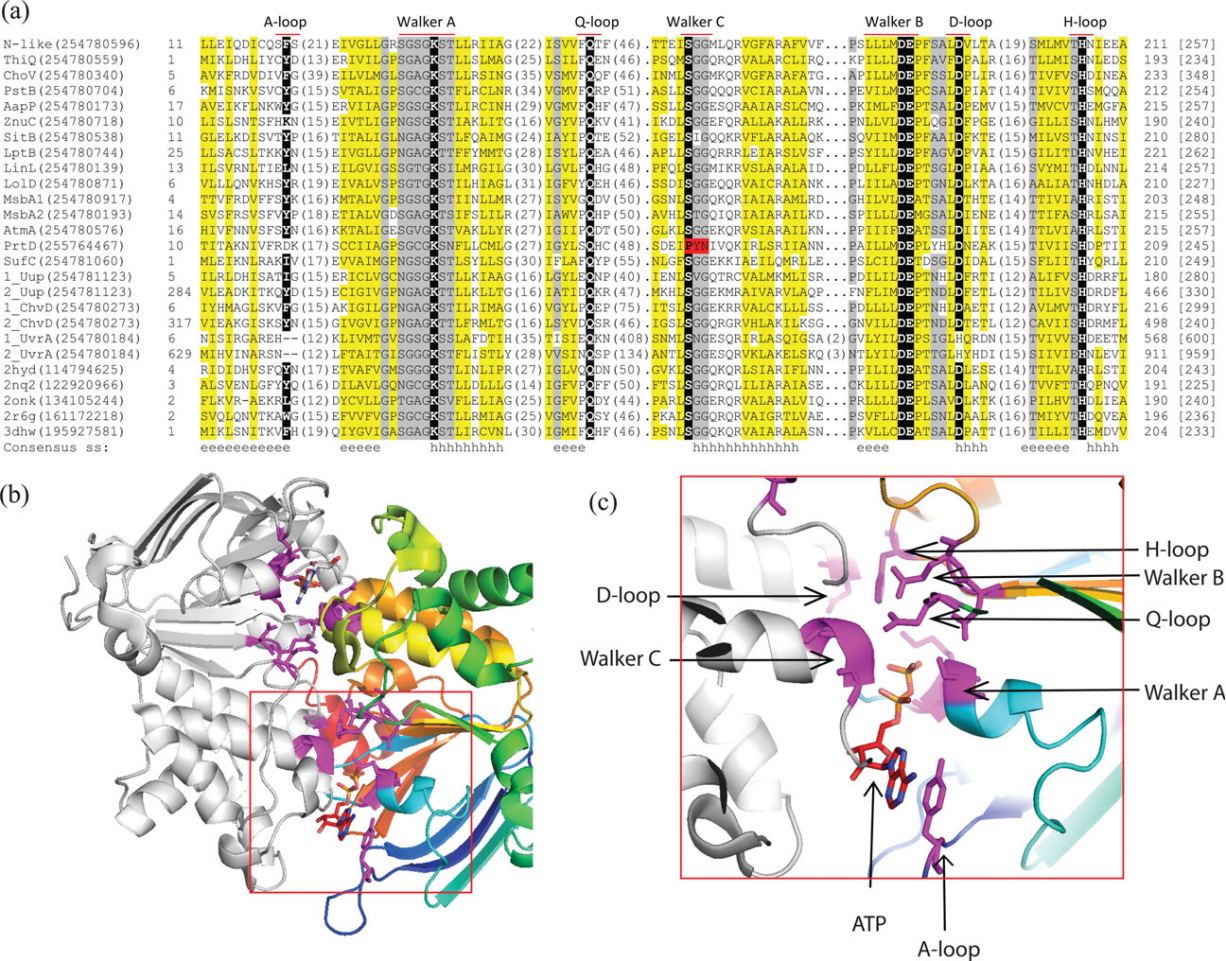
MSA and representative structure of NBDs in *Candidatus* Liberibacter asiaticus proteome. (a) Simplified version of the MSA of all NBDs of *Candidatus* Liberibacter asiaticus and representative homologous structures (only the segments containing sequence motifs of the NBD are shown). The names of motifs are labeled on the top of the MSA. Protein name abbreviations or PDB IDs, with gi number in the parentheses, are used as sequence identifiers at the beginning of each line. N-like is short for Nrt/Ssu/Tau-like system NBD. For ABC-type ATPases with two NBDs, we assign a number in front of the identifier to distinguish between the two domains. In the sequences, hydrophobic residues are highlighted in yellow, small residues positions are colored in gray, and the most essential residues for the function are represented as white letters on black backgrounds. Starting/ending residue numbers and sequence length are shown in italic font and in brackets, respectively. Numbers of residues between the segments are indicated in the parentheses. Dots are used to adjust the space for the MSA. Gaps are shown in dash lines. The PYN residue marked red indicates the substituted Walker C motif. The “consensus ss” line shows the consensus secondary structures predicted by PROMALS3D. For the secondary structure, “e” means beta sheet and “h” stands for alpha helix. (b) Structure of ABC transporter nucleotide-binding domain homodimer with ATP molecules. The structure is adapted from Sav1866 (PDB: 2hyd). The right NBD is colored in rainbow from N to C terminus while the left NBD is colored in gray. Residues essential for the function are shown as sticks in magenta. (c) Close-up of ATP-binding site enlarged from the red frame in (b). ATP and sequence motifs of the NBDs are pointed out.

The NBDs are the most conserved domains among various ABC system proteins. The MSA [Fig 1(a) and Supporting Information Fig. S2] of NBDs from *Ca.* L. asiaticus, including those from the nontransporting ABC proteins, reveals characteristic conservation patterns. To note, three ATPases, that is, MutS, RecF, and RecN, are not included in the MSA due to their diverse sequences. The conserved motifs match known motifs in ABC-type ATPases,^17^ including A-loop, Walker A, Q-loop, Walker C, Walker B, H-loop, and D-loop from the N-terminus to C-terminus, suggesting that the NBDs in *Ca.* L. asiaticus are functional ABC-type ATPases. All these NBDs are evolutionarily related, and their predicted structures all belong to the same family (ABC transporter ATPase domain like) in structural classification of proteins. ABC transporters function as dimers, as shown in Figure 1(b). In the structure, all the sequence motifs are clustered on the interface of the two NBDs [Fig 1(b)]. To bind one ATP molecule, motifs from both sides are involved [Fig 1(c)], allowing the co-ordinate movements of two NBDs upon the binding and hydrolyzing of ATP.^49^ Noticeably, the Walker C motif of PrtD is deteriorated. Whether the substitution disables the function or develops a new functional theme remains to be explored experimentally.

**Figure 2:**
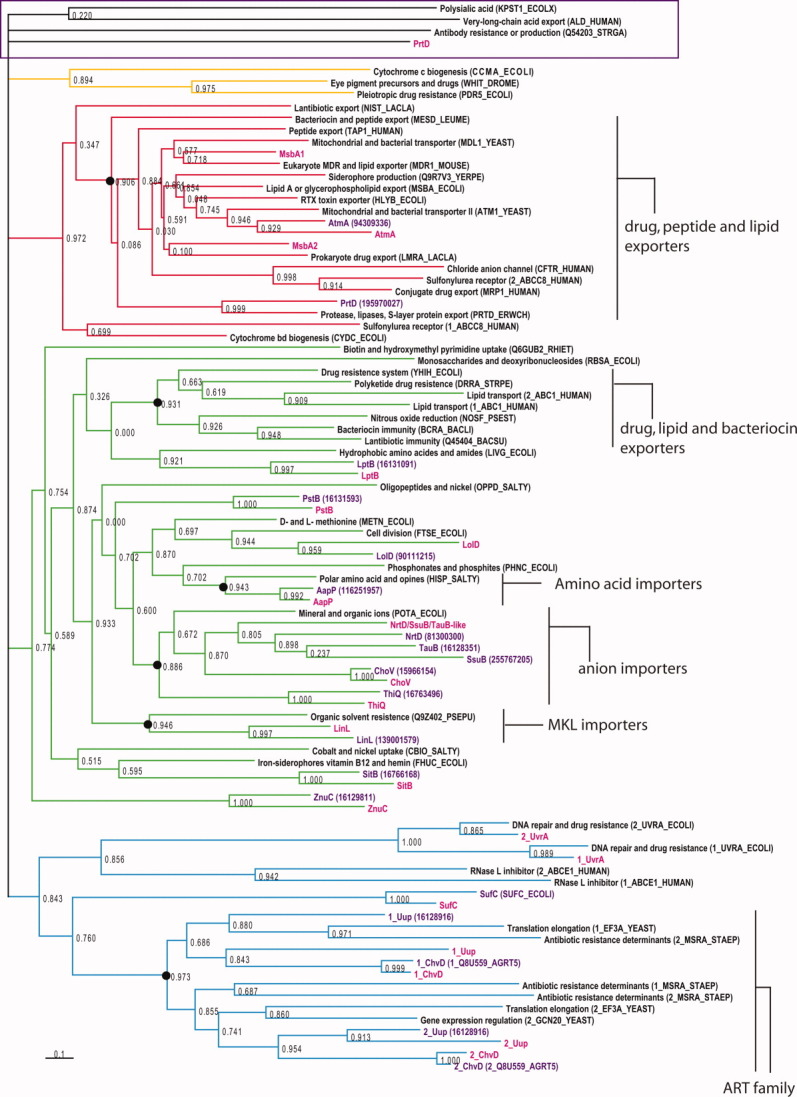
Phylogenetic tree of ABC transporter NBDs. *Ca.* L. asiaticus NBDs and their experimentally studied orthologous NBDs are labeled by the gene abbreviation and colored in pink and purple, respectively. Other NBDs are denoted by their functional definitions. In the parenthesis, gi or unprot id is indicated. For the ABC proteins with multiple NBDs, a domain number is assigned in front of the gene abbreviation as well as the uniprot ID. Bootstrap support values are shown for the internal nodes. The black dots mark the roots of several clades made of proteins with similar substrates. Three major groups are colored in green, red, and blue, respectively. The small group of exporters is colored in orange. The purple box highlights the sequences that are remote from others. ART is short for “antibiotic resistance and translation regulation,” and MKL family represents the family of “retrograde transport of lipids, organic solvent resistance, and steroid uptake.”^15^ The multiple sequence alignment used to generate the tree is available in Supporting Information Figure S9. [Color figure can be viewed in the online issue, which is available at wileyonlinelibrary.com.]

To confirm the close relationships between the *Ca.* L. asiaticus NBDs and their experimentally studied orthologs, we constructed a phylogenetic tree of those NBDs, together with a set of previously analyzed NBDs in Ref.^14^ (Fig 2). Similar to the previous phylogenetic studies,^13^,^14^ the constructed tree topology revealed three major groups colored red, green, and blue, respectively. To note, some nontransport systems (i.e., MutS, RecF, and RecN) are not included due to their diverse sequences. The first major group (red) contains ABC-type exporters mainly for multiple drugs, lipids, peptides, and proteins and corresponds to class I ABC systems in the previous classification. The second major group (green) contains NBDs from both importers (majority) and exporters and corresponds to class III ABC systems in the previous classification. It is possible that these mixed exporters in the second group originated from ancient ABC-type importers and adopted the function of working in efflux systems later in evolution. The third major group (blue) contains mainly nontransport ABC proteins, corresponding to class II ABC systems in the previous classification. Three ATPases (Uniprot ID: CCMA_ECOLI, WHIT_DROME, PDR5_ECOLI) form a small clade (colored orange). The TMDs of the three export systems happen to be in the same clan “ABC-2-transporter-like clan” (CL0181) in the Pfam database while the TMDs of the other exporters (red) are from the clan “ABC transporter membrane domain” (CL0241).

The exhilarating message the phylogenetic tree conveys is that, except for PrtD, all other *Ca.* L. asiaticus proteins are placed closely to the proposed experimentally studied orthologs. Although marginal bootstrap values exist due to the diverse sequences between different ABC-type ATPase families, branches with confident bootstrap values suggest a positive correlation between similarity in substrate preference and similarity in sequence. Six groups with similar substrate preference formed individual clades with good bootstrap probabilities (as indicated by the black dots). However, a few transporters of similar substrates appear to be phylogenetically far from each other. These dispersed branches of similar functions may reveal a real complexity in functional divergence or merely be incorrect tree topology due to nonconfident statistics and insufficient data in the process of evolutionary tree reconstruction. Some sequences placed in long branches (purple frame in Fig 2) are more diverse among the other ATPases. They failed to group with other ATPases possibly due to the insufficient number of representative sequences and long branch attraction problems associated with tree construction.

### Classification of the ABC transporter TMDs in *Ca.* L. asiaticus

**Figure 3:**
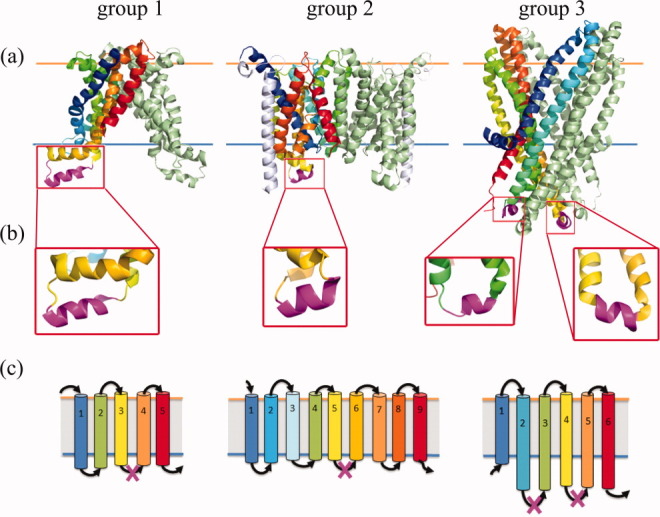
Three groups of ABC transporter TMD structures in *Ca.* L. asiaticus. Representative structure templates for three groups from left to right are 3dhw, 2qi9, and 3b60, respectively. (a) Structure overviews of transmembrane helices. Coupling helices are colored magenta. For each structure dimer, the left TMD is colored from N terminus to C terminus in rainbow; the right TMD is colored in pale green. In Group 2, the residues unaligned by any *Ca.* L. asiaticus sequences are colored in gray. Coupling helix regions are colored magenta and marked in red frames. (b) Enlarged views of the coupling helices. The coupling helices are colored magenta and reorientated for a better view. (c) Topologies of the representative structures colored in rainbow. The coupling helices are marked as magenta crosses. The topology diagram of group 2 does not include the gray N-terminal TMH in the structure. In (a) and (c), the inner membrane region is indicated between the orange and blue lines with the cytoplasm on the bottom. [Color figure can be viewed in the online issue, which is available at wileyonlinelibrary.com.]

In contrast to the conserved NBDs, the TMDs are more divergent in both sequence and structure. For 15 of the 19 TMDs, we were able to generate homology-based structure models and classify them into three groups. Within each group, the TMDs adopt the same fold, and the representative structure templates for these three groups are shown in Figure 3(a). In a recent review, the authors classified solved TMD crystal structures into three different folds,^49^ that is, type I importer, type II importer, and exporter. Nevertheless, the newly established S-component structure of ECF transporter^18^,^19^ exhibited a new fold and thus extended the TMD classification. The three groups of TMDs in *Ca.* L. asiaticus are consistent with these three structure folds in the review^49^ and correspond to three nonhomologous Pfam families (Table I). For each group, the MSAs of the TMDs together with their representative homologs were generated (Supporting Information Figs. S3–S5). Although the sequences appear to be rather diverse, hydrophobic and hydrophilic patterns are preserved. Small residues mediating interhelix interactions and other characteristic residues, such as proline involved in helix kinks, are highly conserved as well.

The coupling helices from different folds exhibit varied sequence features and structures, thus serving as the signature of each fold (Fig 3). TMDs in the first group are from the “binding-protein-dependent transport system inner membrane (IM) component” Pfam family (PF00528), and they adopt the type I importer fold. This group of TMDs possesses five core TMHs, and the essential coupling helix responsible for the interaction between the TMD and NBD is located between the third and fourth TMH [Fig 3(a), left panel]. The coupling helix of the type I importer adopts a semiperpendicular interaction with the following helix [Fig 3(b), left panel]. The short helical pair is connected to their connecting TMHs by kinks. All the TMD sequences in the second group belong to the “ABC 3 transport” family (PF00950) and assume the type II importer fold. Representative type II structures include 10 TMHs [Fig 3(a), center panel]. The type II coupling helix differs from that found in the type I importer fold. It follows a short helix that extends from the sixth TMH by a kink and is connected to the seventh TMH by a short loop [Fig 3(b), center panel]. Compared to the representative structure template consisting of 10 TMHs, the *Ca.* L. asiaticus sequences lack one peripheral TMH at the very N-terminus [colored gray in Fig 3(a) center panel], suggested by HHsearch alignments. As this TMH does not participate in the structure core, its absence should not affect the general fold. All the TMDs in the third group are from “TMD of ABC transporters” family (PF00664) and adopt the exporter fold. They consist of six TMHs that extend into the cytoplasm [Fig 3(a), right panel]. These TMDs form swapped dimers by exchanging the fourth and fifth helices. Upon binding ATP, the TMDs switch from an inward-facing conformation to an outward-facing conformation and release the substrate to the periplasmic space.^50^ Unlike the importers, the exporter fold has two coupling helices [Fig 3(b), right panel]: one is located between the second and the third TMHs and interact with both NBDs in the closed conformation, while the other is located between the swapped fourth and fifth TMHs and inserted into the groove of the NBD on the opposite side.^51^

**Figure 4:**
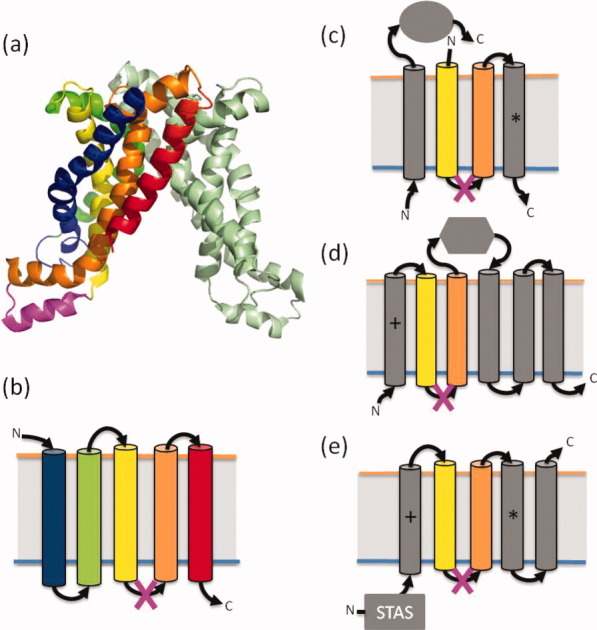
The predicted topology diagram of the TMDs that are related to Pfam clan BPD-like (CL0404). (a) The 3D structure representative (PDB: 3dhw) of TMDs in BPD family (PF00528); (b) the topology diagram of the TMDs in Pfam family BPD (PF00528) colored in rainbow; (c) the topology diagram of the TMD in *Ca.* L. asiaticus Lol system (FtsX family, PF02687); (d) the topology diagram of the TMDs in *Ca.* L. asiaticus Lpt system (YjgP/Q family, PF03739); (e) the topology diagram of the TMD in *Ca.* L. asiaticus Lin system (Permease family, PF02405). In (a), one domain is colored in pale green while the other is colored in rainbow. In (b–e), the cylinders represent the transmembrane helices. Letter N and C indicates the terminus of the proteins. Orange and blue lines define the membrane region with the cytoplasm on the bottom. In (c–e), nontransmembrane domains are represented by filled oval, hexagon, and rectangle, respectively. The coupling helix is colored magenta in the structure and marked by the magenta cross in the topology diagrams. The characteristic helices in HHsearch alignments are colored in yellow and orange. The extended TMHs in the HHsearch alignments between FtsX and Permease, YjgP/Q, and Permease are labeled by asterisks and plus symbols, respectively. [Color figure can be viewed in the online issue, which is available at wileyonlinelibrary.com.]

The other four *Ca.* L. asiaticus TMDs fall into “Permease” (Permease, CLIBASIA_00085), “Predicted Permease YjgP/YjgQ family” (YjgP/Q, CLIBASIA_01390 and CLIBASIA_01395), and “FtsX-like Permease family” (FtsX, peg.788&peg.789) in Pfam. FtsX and YjgP/Q belong to the same Pfam clan “BPD transporter-like” (BPD-like). This suggested homologous relationship is further supported by the pairwise HHsearch probability over 90% (Supporting Information Fig. S6). Moreover, the third Pfam family, Permease, is likely related to FtsX and YjgP/Q families, as suggested by HHsearch (probability over 90%). The suggested sequence relationships are limited to the coupling helix and its surrounding TMHs. The HHsearch alignment between YjgP/Q and Permease extended to the N-terminal TMH (marked by a plus symbol in Fig 4), while the extension in the alignment between FtsX and Permease is one TMH at the C-terminus of surrounding TMHs (marked by asterisk in Fig 4). All three families include a similar predicted minimal transmembrane topology displayed in FtsX [Fig 4(c)]. The presumed core topology includes four helices, with the coupling helix located between the second and third TMH. With respect to this core FtsX TMH topology, YjgP/Q includes an inserted extracellular domain following the third TMH and two additional C-terminal TMHs, and Permease includes an N-terminal cytoplasmic domain and an additional C-terminal TMH. Thus, the three families, FtsX, YjgP/Q, and Permease, share a similar core TMH topology in addition to the type I importer coupling helix motif (HHsearch alignments in Supporting Information Fig S7), suggesting that they adopt similar structures. Intriguingly, the ABC systems consisting of these TMDs in *Ca.* L. asiaticus are all noncanonical transporters. They are involved in shuttling substrates between the IM and the OM, by either releasing (Lol and Lpt) or inserting (Lin) molecules that are lipids or with lipid moieties from/to the outer leaflet of the IM. Such unique and similar transported substrates may serve as evidence to reinforce their relationships.

The Pfam-defined BPD-like clan includes solved structures within the family “Binding-protein-dependent transport system IM component” (PF00528, BPD_transp_1, abbreviated as BPD below). Because proteins in the same Pfam clan indicate that they are evolutionarily related, it raises the question whether the structures of the three families look similar to the known structure in BPD family. The predicted TMD topology of FtsX, YjgP/Q, and Permease differs from the BPD family structure topology (Fig 4). To maintain both the position of the coupling helix and a similar TMH topology, the N-terminal TMH of BPD must be deleted. However, this N-terminal TMH plays an integral role in the BPD fold, maintaining interactions with all other TMHs and positioning the coupling helix (Fig 4). Given the central role of this TMH, its deletion would not likely be tolerated and relating BPD to FtsX would therefore require a less parsimonious pathway of losing the peripheral BPD C-terminal TMH (red), followed by a circular permutation to replace the N-terminal BPD TMH with the C-terminal helix of FtsX. Given this complex requirement for maintaining topology, we could not confidently infer the relationship between the BPD structure and FtsX, bringing into question the Pfam clan assignment.

Another special common feature of the TMDs from Lpt, Lol, and Lin systems is the presence of fused soluble domains (Fig 4). The TMDs of the Lpt system and the Lol system in *Ca.* L. asiaticus are fused with periplasmic domains that likely participate in delivering substrates from the IM to the periplasmic space.^52^ The fused periplasmic domain in the IM proteins (CLIBASIA_01390, CLIBASIA_01395) of the *Ca.* L. asiaticus Lpt system is predicted by HHsearch to be structurally similar to other auxiliary proteins of the *Ca.* L. asiaticus Lpt system, including LptA (CLIBASIA_03160), LptC (CLIBASIA_03165), and one domain in the OM auxiliary protein LptD (CLIBASIA_01400). It is likely that the Lpt system has evolved by duplications to allow efficient conveying of substrates.^53^ The TMD of the Lin system, on the contrary, is fused with a cytoplasmic “Anti-Sigma factor antagonist” (STAS) domain.^54^ In the SulP family transporters,^55^ STAS is suggested to sequester acyl-carrier protein, an essential protein for fatty acid biosynthesis, and thus links transport with fatty acid metabolism. Similarly, the STAS domain in the IM proteins might be able to recruit other proteins and contribute to the regulation of the Lin system or the cross-talks between transporting and other processes.

### Function details of the predicted ABC transporters in *Ca.* L. asiaticus

#### ABC-type importers

In the *Ca.* L. asiaticus proteome, we detected eight ABC-type importers that should be responsible for uptaking essential nutrients from the environment. The substrates of these ABC type importers include amino acids, B family vitamins, ions, and lipids (the first eight systems of Table I). Because it is suggested that *Ca.* L. asiaticus might deplete the host’s nutrient supply, which results in disease symptoms,^8^ these ABC-type importers might help contribute to the death of the plant.

#### Canonical importer systems

The substrate specificities of six nutrient importers can be confidently inferred from their prominent sequence similarity to close homologs with experimentally verified functions. They are general L-amino acid transporter (Aap), phosphate transporter (Pst), thiamine transporter (Thi), choline transporter (Cho), zinc transporter (Znu), and manganese and iron transporter (Sit). One NBD, one PBP, and one or two TMDs are present in each system. Among them, Cho and Znu contain one TMD that should act as a homodimer, and the other four systems contain two homologous TMDs (fused TMDs for Thi system and separated TMDs for others). Because the metabolic pathways for some amino acids are missing in *Ca.* L. asiaticus, the presence of an amino acid transporter (Aap) suggests that this bacterium requires external amino acid supply for its survival. However, we could not deduce the amino acid preference of this transporter, because the orthologous Aap system^56^ in *Rhizobium leguminosarum* is reported to have broad substrate specificity. Thus, understanding the substrate preference of the Aap system experimentally might illuminate the minimal amino acid requirement of *Ca.* L. asiaticus.

Among the six importers, the system with revised annotations is the choline importer (Cho system), which was annotated as a glycine-betaine transporter in existing databases. Although choline and glycine-betaine are chemically similar compounds (glycine-betaine is the oxidized form of choline), the experiments on the orthologous system^57^ in a closely related bacterium *Sinorhizobium meliloti* showed a high specificity for choline, rather than the annotated glycine-betaine substrate. Given the close relationship of the *Ca.* L. asiaticus CLIBASIA_01125 (NBD) component to that in *S. meliloti* as well as the similar operon arrangement (ChoX-ChoW-ChoV), we annotate these components as a Cho system. The highly similar TMD (ChoW, identity: 62%, e-value: 1e^−96^) and PBP (ChoX, identity: 49%, e-value: 5e^−87^) between *Ca.* L. asiaticus and *S. meliloti* further warrant our prediction.

#### A possible novel ABC system

**Figure 5:**
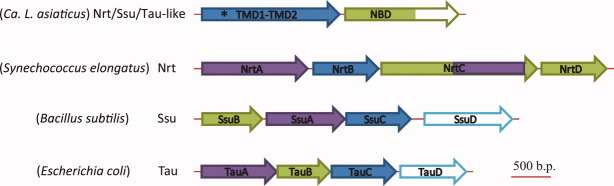
Operon structures of Nrt/Ssu/Tau-like system and its closely related systems. The abbreviation name of each system is shown on the left with the species in the parenthesis. Domain types are shown on the arrows for Nrt/Ssu/Tau-like system while the gene names are shown for the rest three systems. Each gene is shown as an arrow indicating the gene transcription direction. Genes marked with asterisk are proteins with two domains. The NBD, TMD, and PBP are colored green, blue, and purple, respectively. Only NBD, TMD, and PBP are filled. Other operon components not in transporters are denoted in cyan. The PBP in NrtC is a PBP homolog in cytoplasm.^60^ No PBP is detected for Nrt/Ssu/Tau-like system. The scale is illustrated on the bottom right. The three experimental verified systems are from: *Nrt*: *Synechococcus elongatus* PCC 7942^59^; Ssu: *Bacillus subtilis* subsp. subtilis str. 168^61^; Tau: *Escherichia coli* str. K-12 substr. MG1655.^62^ [Color figure can be viewed in the online issue, which is available at wileyonlinelibrary.com.]

The seventh proposed *Ca.* L. asiaticus transporter (Nrt/Ssu/Tau-like system), consisting of one special ATPase containing a characteristic C-terminal domain and another protein containing two TMDs, cannot be classified in terms of a specific substrate. The NBD of the ATPase (CLIBASIA_02415) shows a close relationship to three experimentally studied oxoacid ion transporters, that is, *Synechococcus elongatus* nitrate transporter (Nrt),^58^–^60^*Bacillus subtilis* alkanesulfonate transporter (Ssu),^61^ and *Escherichia coli* taurine transporter (Tau).^62^ However, this Nrt/Ssu/Tau-like system in *Ca.* L. asiaticus shows significantly different component arrangements and protein domain contents from any of the three closely related systems (Fig 5). No PBP has been detected for this system in *Ca.* L. asiaticus, and the membrane component (CLIBASIA_02420) contains two tandem TMDs instead of one TMD in the other three systems. Moreover, the *Ca.* L. asiaticus NBD (CLIBASIA_02415) has a unique fused C-terminal domain that is conserved among a small group of homologs. This additional domain differs from the ABC-type NBD and is classified as “ABC nitrate/sulfonate/bicarbonate family transporter, ATPase subunit” (PF09821), which is not a member of the P-loop NTPase clan (CL0023) in the Pfam database. HHsearch suggests that it adopts a “winged helix” DNA-binding fold with over 95% probability. On the basis of its relatively close relationship to Nrt, Ssu, and Tau, we annotated this system as Nrt/Ssu/Tau-like ABC transporter without a specific functional annotation. The phylogenetic positions of the NBDs (Fig 2) imply that the Nrt/Ssu/Tau-like system might have diverged from the three systems at an early time point. Possibly, the ATPases with this characteristic C-terminal domain may be components of novel ABC transporters that have not been experimentally studied. Given the dramatic differences in its operon organization and the domain structures of the TMDs and the ATPase from the three related systems, whether the Nrt/Ssu/Tau-like system is still a transporter remains questionable and requires further experimental exploration.

#### A noncanonical importer system possibly involved in lipid trafficking

Another special ABC-type transporter in *Ca.* L. asiaticus is the Lin system composed of one NBD, one TMD, and two PBPs. Its closely related protein^63^ in *Sphingobium japonicum* is reported to be involved in the utilization of gamma-hexachlorocyclohexane, presumably by controlling membrane hydrophobicity. However, the detailed mechanism of the Lin transporter in *S. japonicum* remains unclear. Our phylogenetic analysis reveals that the Lin system is closely related to the MKL family of lipid importers.^15^ Experimentally studied systems in the MKL family include phospholipid importer (Mla)^64^ in *Escherichia coli*, cholesterol importer (Mce4)^65^,^66^ in *Mycobacterium tuberculosis*, lipid importer (TGD)^67^ in the chloroplasts of *Arabidopsis*, and a transporter involved in toluene tolerance (Ttg2)^68^*in Pseudomonas putida*. Unlike the other importers that transport substrates across the cell membrane, MKL family importers only insert their substrates, mostly lipid-like compounds, into the IM (Mla and TGD) or cell membrane (Mce4). Inferred from the relationship to MKL family members, the predicted Lin system in *Ca.* L. asiaticus is likely to insert certain lipid components, possibly cargoed from OM like the Mla system, into the IM, thus contributing to the maintenance of membrane hydrophobicity and resistance to organic solvent. Because one canonical transporter should translocate the substrate across the cell membrane, we categorize it as a noncanonical importer.

#### ABC-type exporters

Six ABC-type exporters were detected in the *Ca.* L. asiaticus proteome. Compared to ABC-type importers, exporters generally have a wider spectrum for substrates. The six exporters in *Ca.* L. asiaticus are predicted to contribute mainly to the biogenesis of the OM, multiple drug resistance, and toxin protein secretion.

#### Noncanonical exporters involved in OM biogenesis

The outer membrane (OM) is an essential component of Gram-negative bacteria. To complete the biogenesis of the OM, two types of compounds, that is, lipopolysaccharide (LPS) and lipoprotein, have to get anchored in the OM. The transporting process of these two compounds has two similar steps: first, the precursors anchor into the outer leaflet of the IM and mature to be LPS or lipoprotein in the IM; second, the compounds detach from the outer leaflet of the IM and anchor to the inner leaflet of the OM. For LPS biogenesis, a third step is taken to flip the LPS from the inner leaflet of the OM to the outer leaflet of the OM. Several ABC-type transporters are involved in LPS and lipoprotein translocation.^69^

LPS biogenesis requires two ABC-type exporters, although more than two ABC-type transporters are involved.^69^,^70^ For the first step, lipid A, one of the LPS precursors, is flipped by MsbA.^70^ In *Ca.* L. asiaticus, two copies of MsbA with fused TMD and NBD have been detected. One or both of them could be responsible for this step. The second step involves the Lpt system, consisting of an ABC-type transporter in the IM and a set of auxiliary proteins in the periplasmic space and the OM. The ABC transporter in the *Ca.* L. asiaticus Lpt system includes one NBD, two homologous TMDs, and all other necessary components. For lipoprotein biosynthesis, only one ABC-type transporter (LolD) is involved in the second step of shuttling the substrate between the membranes, while the first step is carried out by Sec translocase.^69^ Compared to the *E. coli* Lol system, the *Ca.* L. asiaticus Lol system, which harbors one NBD, one TMD, and one auxiliary protein, lacks the LolE (TMD) and LolB (auxiliary) genes. This difference is consistent with the previous observation that alphaproteobacteria generally lack LolB and only gammaproteobacteria harbor LolE.^69^ Because LolC (TMD) and LolA (auxiliary) are homologous to LolE and LolB, respectively, it is possible that LolC forms a homodimeric TMD instead of a heterodimer with LolE, and LolA could compensate for the function of LolB.

Unlike other canonical ABC exporters in the proteome, Lpt and Lol systems help substrates to detach from the outer leaflet of the IM, rather than transporting the substrates directly from the cytoplasm to the periplasm.^69^ Thus, Nagao et al.^71^ named these processes “projections” to distinguish those noncanonical exporters. It has been reported that three OM-biogenesis-related systems, namely, Lol, MsbA, and Lpt, are required for the viability of *E. coli*.^72^–^75^ Thus, they could be promising drug targets for Citrus Greening disease treatment.

#### Exporters related to drug resistance

Three *Ca.* L. asiaticus ABC systems with fused NBD and TMD are suggested to be associated with drug resistance. Among them, the NBDs of MsbA1 (CLIBASIA_04080) and MsbA2 (CLIBASIA_00390) show high pairwise identity (43%) to each other, and both show high identity to *E. coli* MsbA (Table I). Although their substrate preferences might differ, the experimental studies on this family of proteins do not provide enough information to distinguish their substrates. Knowing that the *E. coli* MsbA, the proposed ortholog for *Ca.* L. asiaticus MsbA1 and MsbA2, is capable of generating multidrug resistance,^76^ we proposed that those two copies of MsbA could also be involved in exporting multiple drugs.

Another *Ca.* L. asiaticus system, whose NBD is fused with its TMD and shows 45% identity to that of *Ca.* L. asiaticus MsbA1, is the AtmA exporter (CLIBASIA_02315). Its close homolog (AtmA)^77^ in *Cupriavidus metalliduran* functions as a transporter that is related to cobalt and nickel resistance. Therefore, it is likely that the *Ca.* L. asiaticus AtmA also functions in heavy metal resistance, possibly by exporting heavy metals.

#### A type I secretion system in Ca. L. asiaticus

A special ABC-type exporter in *Ca.* L. asiaticus is the type I secretion system (PrtD). It is one of only two protein secretion systems (the other is the Sec protein secretion system) present in *Ca.* L. asiaticus.^8^ Type I secretion systems can export proteins of varied sizes and are responsible for secreting RTX (repeat in toxin) proteins.^78^,^79^ Although *Ca.* L. asiaticus PrtD (CLIBASIA_01350) has been annotated as a type I secretion system ATPase in the current database, its low-sequence identity to the experimentally studied ortholog (27%) and the substitution of Walker C motif (Fig 1) question its function. One explanation for the low similarity might be that the *Ca.* L. asiaticus PrtD NBD exhibits an elevated evolutionary rate compared to the other type I secretion systems, as suggested by the distant relationships to its homologous type I secretion systems in CLANS clusters (Supporting Information Fig. S8). More importantly, the presence of a highly characteristic type I secretion system substrate, RTX protease serralysin^79^,^80^ (CLIBASIA_01345, NCBI gi: 254780384) next to the *Ca.* L. asiaticus PrtD NBD, suggests that this type I secretion system should be capable of exporting substrates, at least RTX proteases. Because the transporting cycle requires energy provided by ATP hydrolysis, the Walker C substitution of this possibly functional type I secretion system would be an intriguing issue to investigate in terms of the structure, function, and co-operativity between two NBDs. In ABCG5-ABCG8 heterodimeric sterol transporter, an intact Walker A and Walker B from ABCG5 functions together with an intact Walker C from ABCG8 that is essential for transport activity. The second nucleotide-binding site is deteriorated, and substitution of Walker C in ABCG5 does not affect the sterol secretion.^81^ Thus, it is possible that the *Ca.* L. asiaticus PrtD with the deteriorated Walker C developed a partnership with other NBDs and only contributes its Walker A and Walker B to form the ATP-binding site.

### Nontransport ABC proteins

Seven nontransport ABC-type ATPases are detected in *Ca.* L. asiaticus. Although not involved in transporting, they are related to important cellular processes such as Fe-S assembly (SufC),^82^ virulence gene regulation (ChvD),^83^ transposon excision regulation (Uup), and DNA repair regulation (UvrA, MutS, RecF, and RecN).^15^ The four ATPases involved in DNA repair show diverse sequence features compared to the ABC-type ATPases. For three of them (UvrA, RecF, and RecN), a long insertion between the Q-loop and Walker C motif makes it difficult to detect their relationships to ABC-type ATPases, while the Q-loop and Walker C motif are lacking in the MutS sequence. We also detected a short *Ca.* L. asiaticus protein (CLIBASIA_02635, 110 residues, not listed in Table I) similar to the N-terminal region of Rad50 (1312 residues in *Saccharomyces cerevisiae*), a structure maintenance of chromosome family protein. This protein may be a relic of evolution.

The novel *Ca.* L. asiaticus nontransport ATPase is now annotated as Uup. The closest experimentally studied ortholog is the *E. coli* Uup (gi: 16128916), a soluble protein involved in transposon excision regulation.^84^–^87^ Although the overall identity of *Ca.* L. asiaticus Uup to *E. coli* Uup is marginal (35%), possibly due to the different insertion length between the Q-loop and Walker C motif in the first NBD, its homologous relationship is supported by the high sequence similarity of the second NBD (about 49%). Another piece of evidence is a coiled-coil domain in the C-terminus of the *Ca.* L. asiaticus Uup predicted by COILS,^88^ which is consistent with the presence of a similar coiled-coil domain at the C-terminus of *E. coli* Uup. This coiled-coil domain in *E. coli* Uup is essential for overall structure stability and participates in binding DNA.^89^ Therefore, similar to *E. coli* Uup, the *Ca.* L. asiaticus Uup might also have the ability to regulate the DNA excision. A recent gene deletion study also proposes the Uup protein to be involved in bacterial quorum-sensing^90^ mediated by direct contact between the cells, which makes this predicted Uup an interesting target for experimental study as the quorum-sensing phenomenon is thought to play an important role in bacterial virulence.^91^

### Incomplete systems

Five ABC-system proteins in *Ca.* L. asiaticus do not have confident NBD partners (Supporting Information Table SI). Considering the small genome size of this bacterium, these proteins might be the evolutionary relics of genome reduction. Alternatively, these “orphan” ABC system proteins, either TMD or PBP, may be able to adopt functions other than ABC transport. A recent study^92^ in *Arabidopsis* ABCG family transporters reported that ABCG11 could form a homodimer with itself or a heterodimer with ABCG12. Considering the promiscuous dimerization of ABC transporter in *Arabidopsis*, it is also possible that the ATPases from complete systems could exhibit multiple functions and might be capable of forming a functional ABC-transporter with these “orphan” components.

## CONCLUSIONS

Combining various computational methods, we identified a complete set of ABC transporters and several other nontransport ABC systems in the *Ca.* L. asiaticus proteome, confirmed annotations for most of the ABC system proteins, predicted the polarity and structure of each ABC transporter, and generated new annotations for seven proteins from four ABC systems. Although the features of most ABC systems could be deduced from the abundant experimental data on their orthologs, we reported several novel observations, including a Nrt/Ssu/Tau-like transporter that has never been studied, a deterioration of the Walker C motif in the type I secretion system, the duplication events in the periplasmic components of the Lpt system, and the remote homology relationships between the FtsX, YjgP/Q, and Permease Pfam families. In addition, our analysis reveals several proteins likely important for controlling the Citrus Greening disease, including the type I secretion system and its substrate, and the essential ABC transporter systems involved in bacterial OM biosynthesis. Further studies targeting these proteins might lead to better understanding and treatment of HLB.
