# Supplementary figures and images for: The ABC transporters in Candidatus Liberibacter asiaticus
Source: Proteins. 2012 Jul 31;80(11):2614–28. doi: 10.1002/prot.24147 (PMC3688454; doi:10.1002/prot.24147)

Worst -----Best INFO: blast=blastall -p blastp refdb=

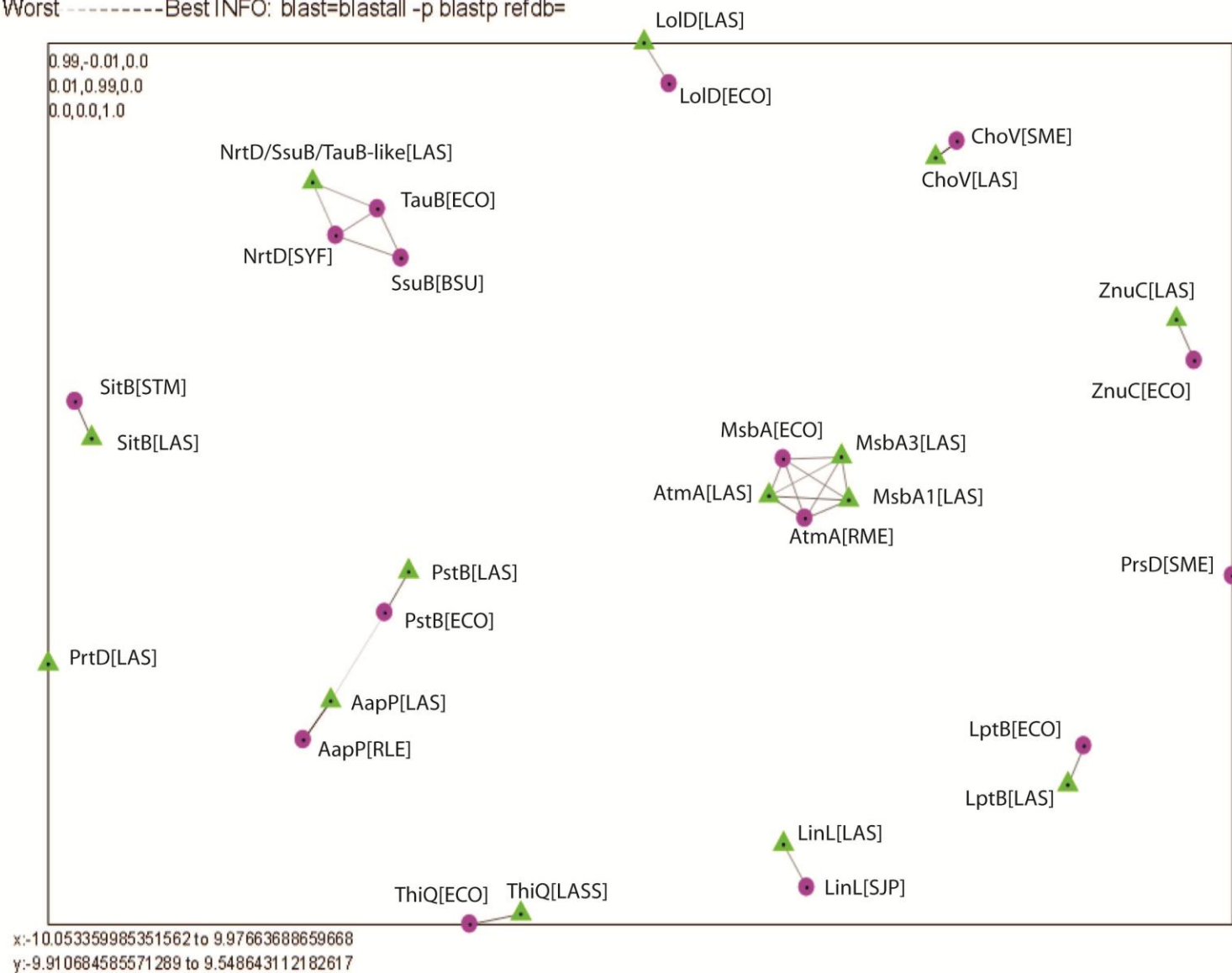

FIGURE S1

Supplement: Supplementary file 1 [file prot0080-2614-sd1.pdf]

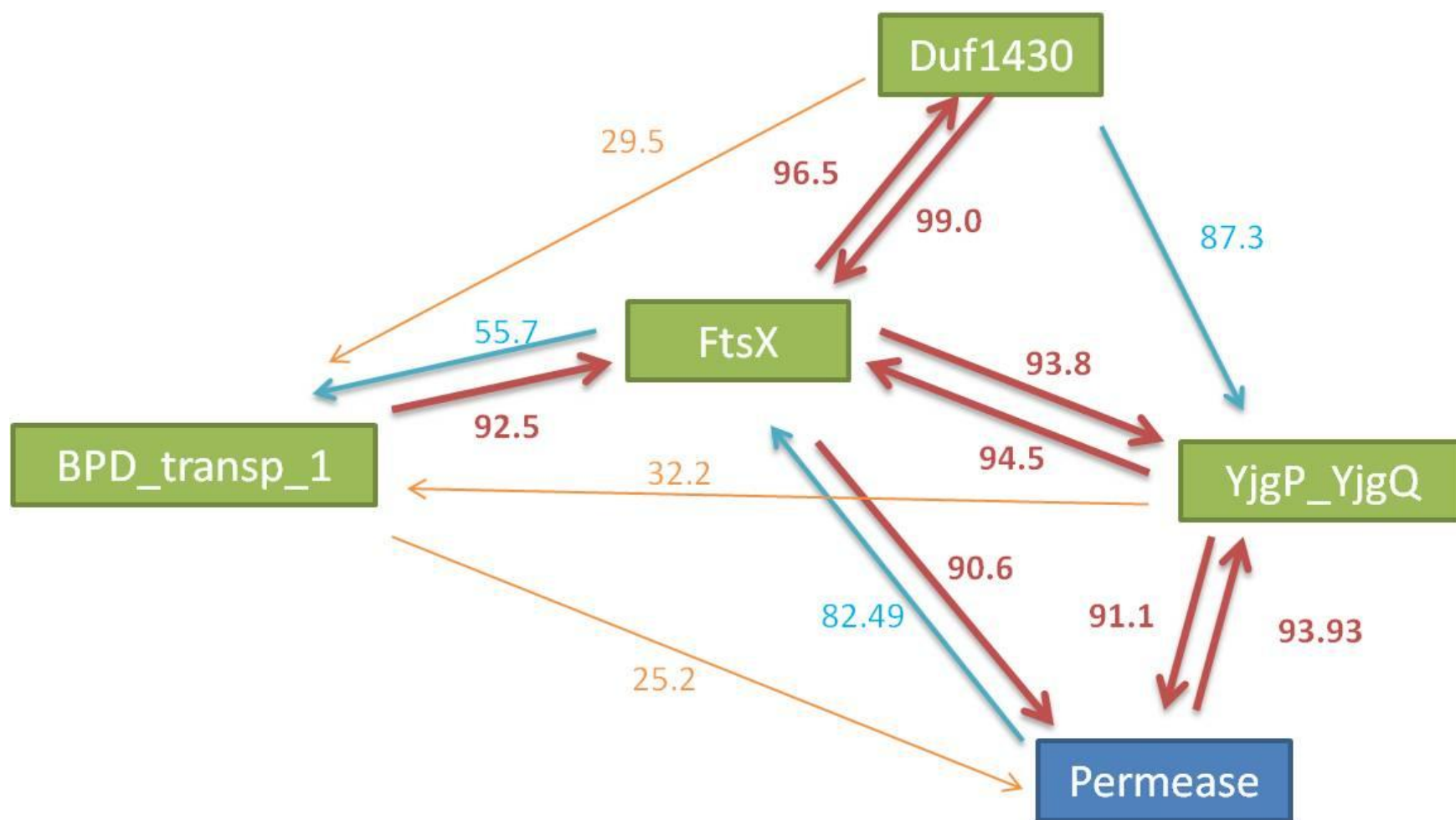

FIGURE S6

Supplement: Supplementary file 6 [file prot0080-2614-sd6.pdf]

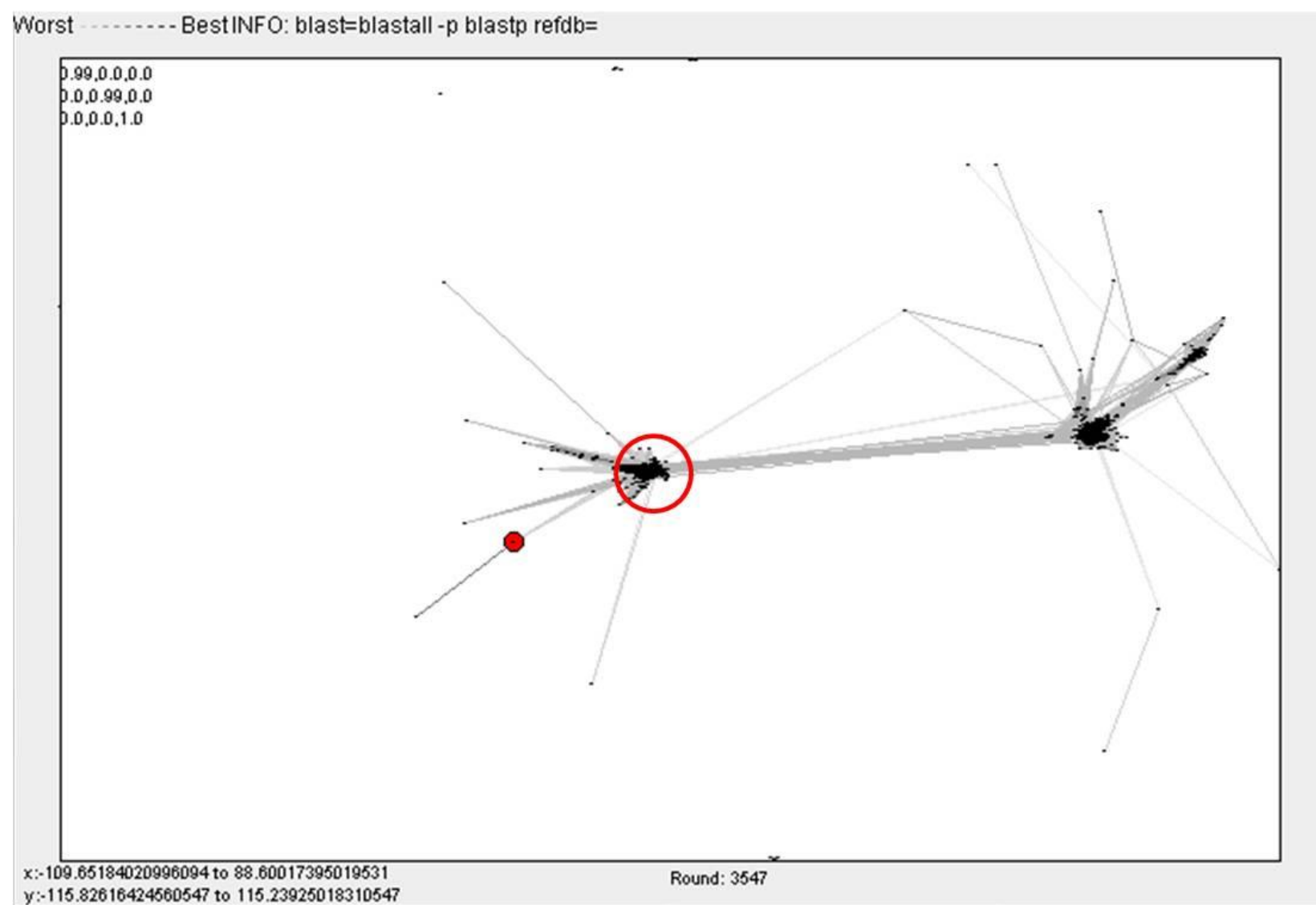

FIGURE S8

Supplement: Supplementary file 8 [file prot0080-2614-sd8.pdf]
