# Supplementary material for: The ABC transporters in Candidatus Liberibacter asiaticus
Source: Proteins. 2012 Jul 31;80(11):2614–28. doi: 10.1002/prot.24147 (PMC3688454; doi:10.1002/prot.24147)
Supplement: Supplementary file 5 [file prot0080-2614-sd5.pdf]

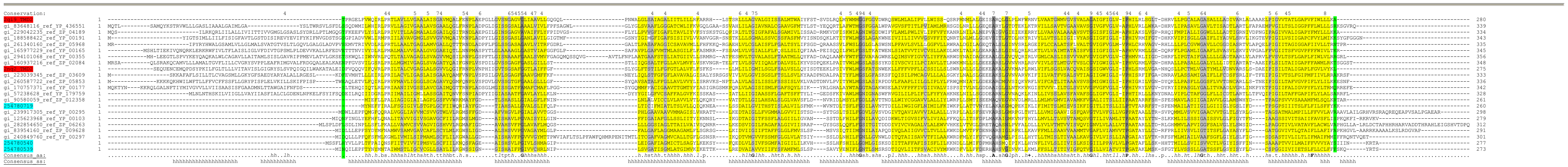

FIGURE S5
