# Supplementary material for: The ABC transporters in Candidatus Liberibacter asiaticus
Source: Proteins. 2012 Jul 31;80(11):2614–28. doi: 10.1002/prot.24147 (PMC3688454; doi:10.1002/prot.24147)
Supplement: Supplementary file 7 [file prot0080-2614-sd7.pdf]

FIGURE S7

3dhw against FtsX

```
>PF02687 FtsX:  FtsX-like permease family;  InterPro: This entry represents an uncharacterised domain
  that is found in ABC transporter permeases.; GO: 0016020 membrane
  Probab=92.45  E-value=2  Score=29.16  Aligned_cols=121  Identities=17%  Similarity=0.099  Sum_probs=0.0

  Q ss_pred             HHHHHHHHHHHHHHHHHHHHHHhCCHHHHHHHHHcCCCHHHHHHHHHHHHHHHHHHHHHHHHHHHHHHHHHHHHHHHHHcccH
  Q Thu_Jun_23_19:   92 AIVPLTVGAAPFIARMVENALLEIPTGLIEASRAMGATPMQIVRKVLLPEALPGLVNAATITLITLVGYSAMGGAVGAGG
171 (217)
  Q Consensus        92 ~il~~~~~~~~~~~~~~~~~~~~i~~~~~~aa~~~G~s~~~~~~~~v~lP~~~~~il~~~~~~~~~~~~~~~~~~~~~~lg~~g
171 (217)
                        .+....................+-+++-...-|..|+|++|+.+.+..-...........-.....+........+...
  T Consensus         2 i~~~l~~~i~~~~~~~~~~~~~~~~~r~~~i~il~~~G~s~~~i~~~~~~~e~~~~~~~~~~~i~g~~~~~~~~~~~~~~~~~~~~~~-
80 (122)
  T PF02687_consen    2 ILAVLILLIAIFVLYNTIRMSIQERKREIAILRALGASKKQIIRMLLIEALLIALIAIILGLILGFLLSWLFYRAFFSI-
80 (122)
  T ss_pred             HHHHHHHHHHHHHHHHHHHHHHHHHHHHHHHHHHcCCCchhhHHHHHHHHHHHHHHHHHHHHHHHHHHHHHHHHHHHHHHh-


  Q ss_pred             HHHHHHHHhhccccHHHHHHHHHHHHHHHHHHHHHHHHHHHHHHhcC
  Q Thu_Jun_23_19:  172 LGQIGYQYGYIGYNATVMNTVLVLLVILVYLIQFAGDRIVRAVTR  216 (217)
  Q Consensus       172 ~g~~~~~~~~~~~~~~~~~~~~~~~~~~~~~~~~~~~~~~~~~l~l~l~~~~~~~~~l~~~l~~~~~~~~  216 (217)
                           ..........+++..........++........-+--+|.+|
  T Consensus        81 ---~~~~~~~~~~~~~~~~~~~~~~~~~~~~~~~~~~~~~~~~~~~~~~~~~~~l~~~~~~~~~~~~~~~~~~r~~P  122 (122)
  T PF02687_consen   81 ---DFIFSLPISLPPWALLLIFVLILLLFLLASLIPARKILRISP  122 (122)
  T ss_pred             ---ccccccceeeeCHHHHHHHHHHHHHHHHHHHHHHHHHHHCCCC
```

FtsX against DUF1430

```
>PF07242 DUF1430:  Protein of unknown function (DUF1430);  InterPro: These sequences represent a family
```

of integral membrane proteins, most of which are about 650 residues in size and predicted to span the membrane seven times. Nearly half of the members of this family are found in association with a member of the lactococcin 972 family of bacteriocins (IPR006540 from INTERPRO) []. Others may be associated with uncharacterised proteins that may also act as bacteriocins. Although this protein is suggested to be an immunity protein, and the bacteriocin is suggested to be exported by a Sec-dependent process, the role of this protein is unclear.

```
 Probab=96.49  E-value=0.05  Score=35.07  Aligned_cols=62  Identities=19%  Similarity=0.293  Sum_probs=0.0

 Q ss_pred              HHHHHHHHHHHHHHHHHHHHHHHHHHHHHHHhCCCHHHHHHHHHHHHHHHHHHHHHHHHHHH
 Q Thu_Jun_23_18:   21 IGLVFTAMFLISNTIKITIFARRKEIEIMKLVGATNWFIRWPFFLEGLLLGVFGSVIPIALV   82 (140)
 Q Consensus        21 ~~~~~ia~~~i~~~~~~~~i~~r~~ei~ilk~lG~~~~~i~~~~~~e~~~l~~~g~~lg~~~~   82 (140)
                       .+.++...+.+..+....-+++|+|+.+.|..|++-.+..+.++.........+..+.....
 T Consensus         1 Ii~l~~~ili~~~~~~lYFe~~kk~i~Ik~i~Gys~f~~hk~yl~~~~~~~i~~~~~~~i~~   62 (100)
 T PF07242_consen    1 IITLISSILIIYQMNLLYFEQNKKKIAIKKIFGYSFFKRHKRYLLSQLISWILGLIIAFILS   62 (100)
 T ss_pred              ChHHHHHHHHHHHHHHHHHHHHHHHHHHHHHhCchHHHHHHHHHHHHHHHHHHHHHHHHHHH
```

FtsX against YjgP/Q

>PF03739 YjgP_YjgQ: Predicted permease YjgP/YjgQ family; InterPro: Members of this family are predicted integral membrane proteins of unknown function. They are about 350 amino acids long, contain about 6 transmembrane regions and may be permeases, although there is no verification of this.; GO: 0016021 integral to membrane

```
 Probab=93.78  E-value=0.69  Score=35.61  Aligned_cols=71  Identities=14%  Similarity=0.124  Sum_probs=0.0

 Q ss_pred              HHHHHHHHHHHHHHHHHHHHHHHHhCCCHHHHHHHHHHHHHHHHHHHHHHHHHHHHHHHHHHHHHHHHhhh
 Q Thu_Jun_23_18:   28 MFLISNTIKITIFARRKEIEIMKLVGATNWFIRWPFFLEGLLLGVFGSVIPIALVLSTYQYVIGWVVPKVQ   98 (140)
 Q Consensus        28 ~~~i~~~~~~~~i~~r~~ei~ilk~lG~~~~~i~~~~~~e~~~l~~~g~~lg~~~~~~~~~~~~~~~~~~~~~~   98 (140)
                       .+.+.......-.++.+|+-.+|+.|.+.+++.++++.-+.+++++....+..+.-...........+..+
 T Consensus        63 a~lla~l~~~~~L~~~~El~a~~asGiS~~~i~~p~l~~~~~i~~~~~~~~~~~~~v~P~~~~~~~~~~~~~~~~~~~  133 (354)
 T PF03739_consen   63 AVLLAVLLTLGRLSRNNELTAMRASGISLKRILRPILIFALLISIIMFILNEWVAPWANKQAEEIRSEIRQ  133 (354)
 T ss_pred              HHHHHHHHHHHHHHhcchHHHHHHcCCCHHHHHHHHHHHHHHHHHHHHHHhchHHHHHHHHHHHHHHHHHh
```

FtsX against DUF140

```
 >PF02405 DUF140:  Domain of unknown function DUF140;  InterPro: This domain has no known function nor
  do any of the proteins that possess it. The aligned region is approximately 150 amino acids long.
  Probab=80.52  E-value=17  Score=26.46  Aligned_cols=112  Identities=12%  Similarity=0.119  Sum_probs=0.0

  Q ss_pred                  HHHHHHHHHHHHHHHHHHHHHHHHHHHHHHHHHHHhCCCHHHHHHH-----HHHHHHHHHHHHHHHHHHHHHHHHHHHHHHH
  Q Thu_Jun_23_18:   19 LIIGLVFTAMFLISNTIKITIFARRKEIEIMKLVGATNWFIRWP-----FFLEGLLLGVFGSVIPIALVLSTYQYVIGWV
93 (140)
  Q Consensus        19 ~~~~~~~ia~~~i~~~~~~~~i~~r~~ei~ilk~lG~~~~~i~~~-----~~~e~~~l~~~g~~lg~~~~~~~~~~~~~~~
93 (140)
                         +....++.+=.......-....+-++|+.-+|++|.++.+-.-.     ...-.-.+...+...|+..|+.......+.-
  T Consensus        61 ~ltali~agR~GsaiaAElG~M~v~eqIDAL~~mgi~P~~yLv~PRi~A~~i~~p~L~~~~~~~~~~~~gg~~~~~~~~~~~
140 (215)
  T PF02405_consen   61 VLTALILAGRVGSAIAAELGTMRVTEQIDALEVMGIDPIRYLVVPRILAMVIMMPLLTIIFDLVGILGGYLVAVFFLGIS
140 (215)
  T ss_pred                  HHHHHHHHHHHhhHHHHHHHHHHHhHHHHHHHHcCCChHHHHHHHHHHHHHHHHHHHHHHHHHHHHHHHHHHHHHHhcCCC


  Q ss_pred                  HHhhhhhhhccCChHHHHHHHHHHHHHHHHHHHHHHHHH
  Q Thu_Jun_23_18:   94 VPKVQGSFVSLLPYNPFVFQVSLVLIAIGAVIGVWGSL  131 (140)
  Q Consensus        94 ~~~~~~~~~~~~~~~~~~~~~~~~~~~~~~~~l~~~~~a~  131 (140)
                         ...|-.+.....++.++ +....-....+..+++++.+
  T Consensus       141 ~~~f~~~~~~~~~~~~dl-~~~l~K~~vFg~~i~~i~cy  177 (215)
  T PF02405_consen  141 PGAFLQSFRSFLTPKDL-LVGLIKAAVFGLIIALIACY  177 (215)
  T ss_pred                  HHHHHHHHHHHCCHHHH-HHHHHHHHHHHHHHHHHHHHH
```

Duf1430 against FtsX

```
>PF07242 DUF1430:  Protein of unknown function (DUF1430);  InterPro: These sequences represent a family
  of integral membrane proteins, most of which are about 650 residues in size and predicted to span the
membrane
  seven times. Nearly half of the members of this family are found in association with a member of the
```

```
  lactococcin 972 family of bacteriocins (IPR006540 from INTERPRO) []. Others may be associated with
uncharacterised
  proteins that may also act as bacteriocins. Although this protein is suggested to be an immunity protein,
and the
  bacteriocin is suggested to be exported by a Sec-dependent process, the role of this protein is unclear.
  Probab=99.85  E-value=1e-20  Score=168.79  Aligned_cols=100  Identities=34%  Similarity=0.566
Sum_probs=0.0

  Q ss_pred               HHHHHHHHHHHHHHHHHHHHHHhHHhhHHHHHhCcCHHHHHHHHHHHHHHHHHHHHHHHHHHhhHHHHHHHHHHHHHHHH
  Q Thu_Jun_23_22:  619 IILAISSYSFMILLVLSYYRENLYKQLIYHVFGYSFWKSSKWFIISNLAVCAFSGVVTFIITKEPIALYFSVVILLIELC
698 (722)
  Q Consensus       619 ii~iis~lil~~~~~~~~Yf~~~rk~i~ik~l~G~sf~~~~k~~l~~~~~~~~~~~~~~~~~~~~~~~~i~~~~l~~lileli
698 (722)
                        |++++++++++++++.+|||+|||++++||++|||+|+|||+|+.++++++++++++++++++++++++++++++++++|.+
  T Consensus         1 Ii~l~~~ili~~~~~~lYFe~~kk~i~Ik~i~Gys~f~~hk~yl~~~~~~~~i~~~~~~~~i~~~~~~~~~~l~~~~~~i~e~~
80 (100)
  T PF07242_consen    1 IITLISSILIIYQMNLLYFEQNKKKIAIKKIFGYSFFKRHKRYLLSQLISWILGLIIAFILSKNIIILLIILFLFIIELL
80 (100)
  T ss_pred               ChHHHHHHHHHHHHHHHHHHHHHHHHHHHHHhCchHHHHHHHHHHHHHHHHHHHHHHHHHHHHhhHHHHHHHHHHHHHHHH


  Q ss_pred               HHHHHHHHHHHHHHHHHhcC
  Q Thu_Jun_23_22:  699 VIYFLKEKVINKDFKAILKG  718 (722)
  Q Consensus       699 ~~~~~i~~~ekk~~~~ilKG  718 (722)
                        ++++..++.|+|+++++|||
  T Consensus        81 ~~l~~~~~~~e~k~~~~vLKG  100 (100)
  T PF07242_consen   81 LILLIIMKKEKKNFISVLKG  100 (100)
  T ss_pred               HHHHHHHHHHhhhHHHHhCc
```

YjgP/Q against FtsX

>PF02687 FtsX: FtsX-like permease family; InterPro: This entry represents an uncharacterised domain

```
  that is found in ABC transporter permeases.; GO: 0016020 membrane
  Probab=94.55  E-value=0.52  Score=32.25  Aligned_cols=76  Identities=12%  Similarity=0.171  Sum_probs=0.0

  Q ss_pred              HHHHHHHHHHHHHHHHHHHHHhcCCcHHHHHHcCCCHHHHHHHHHHHHHHHHHHHHHHHhChHHHHHHHHHHHHHH
  Q Thu_Jun_23_18:   81 DIEIFFPMAALLGALLGLGTLAQRSELVVMQAAGFTRMQVALSVMKTAIPLVLLTMAIGEFVAPQGEQMARNFRAQ  156
(160)
  Q Consensus        81 ~l~~~lP~a~llA~i~~~~~L~~~~El~a~~asGiS~~~i~~p~l~~~l~i~~~~~~~~~~~~i~P~s~~~~~~i~~~  156
(160)
                         .....+=..+.+.......--.|.+|+..+|+.|.|.+++.+..+.-+...+.+....+..+.-...+-.++.-.+
  T Consensus         4 ~~l~~~i~~~~~~~~~~~~~~~~~~r~~~i~il~~~G~s~~~i~~~~~~~e~~~~~~~~~~~~i~g~~~~~~~~~~~~~~~~~~~~   79
(122)
  T PF02687_consen    4 AVLILLIAIFVLYNTIRMSIQERKREIAILRALGASKKQIIRMLLIEALLIALIAIILGLILGFLLSWLFYRAFFS   79
(122)
  T ss_pred              HHHHHHHHHHHHHHHHHHHHHHHHHHHHHHHHcCCChhhHHHHHHHHHHHHHHHHHHHHHHHHHHHHHHHHHHHHHHH
```

YjgP/Q against DUF140

```
>PF02405 DUF140:  Domain of unknown function DUF140;  InterPro: This domain has no known function nor
  do any of the proteins that possess it. The aligned region is approximately 150 amino acids long.
  Probab=91.11  E-value=5.5  Score=31.38  Aligned_cols=108  Identities=12%  Similarity=0.107  Sum_probs=0.0

  Q ss_pred              HHHHHHHHHHHHHHHHHHHHHHHHH-HhCCCCHHHHHHHHHHHHHHHHHHHHHHHHHHHHHHHHHHHHHHhcCCcHHHHHHcC
  Q Thu_Jun_23_18:   36 TILMTLFMLVSLSGIIKFVDQLRKT-GQGAYSALDAGYYTLLSVPKDIEIFFPMAALLGALLGLGTLAQRSELVVMQAAG
114 (160)
  Q Consensus        36 ~f~~~l~~~~~~i~~~~~~l~~~l~~~-~~~~~~~~~~~~il~~~~~~~lP~~l~~~lP~a~llA~i~~~~~L~~~~El~a~~asG
114 (160)
                         +.......+.+.....|...+++++ .+.-.......-..-=.-|..-...+---.-=|..--++.|.-+.|+.|+++-|
  T Consensus        16 ~iv~l~~~~~~G~v~a~q~~~~l~~~G~~~~vg~~~~~~~~~vrEl~P~ltali~agR~GsaiaAElG~M~v~eqIDAL~~mg
95 (215)
  T PF02405_consen   16 PIVLLTSFFIGLVLALQGAYQLRRFGAESLVGSIVGLAIVRELGPVLTALILAGRVGSAIAAELGTMRVTEQIDALEVMG
95 (215)
```

```
  T ss_pred             HHHHHHHHHHHHHHHHHHHHHHHHhChHHHHHHHHHHHHHHHHHHHHHHHHHHHHHhhHHHHHHHHHHHHhHHHHHHHcC


  Q ss_pred             CCHHHHHH-HHHHHHHHHHHHHHHHhChHH
  Q Thu_Jun_23_18:  115 FTRMQVAL-SVMKTAIPLVLLTMAIGEFVA  143 (160)
  Q Consensus       115 iS~~~i~~-p~l~~~l~i~~~~~~~~~~~~i~  143 (160)
                        ++|.+-+- |=++.+.+.........+.+.
  T Consensus        96 i~P~~yLv~PRi~A~~i~~p~L~~~~~~~~~  125 (215)
  T PF02405_consen   96 IDPIRYLVVPRILAMVIMMPLLTIIFDLVG  125 (215)
  T ss_pred             CChHHHHHHHHHHHHHHHHHHHHHHHHHHH
```

DUF140 against YjgP/Q

```
>PF03739 YjgP_YjgQ:  Predicted permease YjgP/YjgQ family;  InterPro: Members of this family are
  predicted integral membrane proteins of unknown function. They are about 350 amino acids long, contain
about 6
  transmembrane regions and may be permeases, although there is no verification of this.; GO: 0016021
integral to
  membrane
  Probab=93.93  E-value=0.97  Score=41.98  Aligned_cols=103  Identities=18%  Similarity=0.182
Sum_probs=0.0

  Q ss_pred             HHHHHHHHHHHHHHHHHHHHHHHHhChHHHHHHHHHHHHHHHHHHHHHHHHHHHHHhhHHHHHHHhhhHhhHHHHHHHHcC
  Q Thu_Jun_23_23:   82 PVVILISFVTGAVIAQQGAFQLSQFGAEIFSIDLMSILQLREIGVLLTAVMIAGRSGSAIVAEIGSMKINEEIDAIRTMG
161 (286)
  Q Consensus        82 piv~l~a~~iG~vl~~Q~~~~l~~fGa~~~vg~~~~~~ivrEl~PlltalllagR~GsaiaAElGtMrvteqIDAL~~mg
161 (286)
                        +....+..+++..+..|....++.+ .+.-.+.....-..-=.-|-....++-.=+=-|...-+|.|.-+.|+-||++.|
  T Consensus        10 ~f~~~~~~~~~l~~i~~~~~~l~~~-~~~~~~~~~i~~~~~~~lP~~l~~~lP~a~lla~l~~~~~L~~~~El~a~~asG
88 (354)
  T PF03739_consen   10 PFLLVLLSFVGLFFIIDLLDLLDDF-LSSGLSLSDILKYILYQLPYFLYYILPIAVLLAVLLTLGRLSRNNELTAMRASG
88 (354)
  T ss_pred             HHHHHHHHHHHHHHHHHHHHHHHHH-HcCCCCHHHHHHHHHHHHHHHHHHHHHHHHHHHHHHHHHHHHhcchHHHHHHcC
```

```
Q ss_pred                CChhHHhhHHHHHHHHHHHHHHHH
Q Thu_Jun_23_23:  162 LDFVRILISPRIWALIISLPLLTI  185 (286)
Q Consensus       162 idP~~yLV~PRilA~vi~~p~L~~  185 (286)
                       ++|.+-+.-+-+++.+++...+..
T Consensus        89 iS~~~i~~p~l~~~~~i~~~~~~~  112 (354)
T PF03739_consen   89 ISLKRILRPILIFALLISIIMFIL  112 (354)
T ss_pred                CCHHHHHHHHHHHHHHHHHHHHHH
```
