# Supplementary material for: The ABC transporters in Candidatus Liberibacter asiaticus
Source: Proteins. 2012 Jul 31;80(11):2614–28. doi: 10.1002/prot.24147 (PMC3688454; doi:10.1002/prot.24147)
Supplement: Supplementary file 10 [file prot0080-2614-sd10.pdf]

Table SI. ABC system proteins in *Ca. L. asiaticus*.

System name	NBD	TMD	Other components	Function prediction	Polarity	TCDB
	**GI | protein name**	**GI | Pfam clan (Pfam clan ID) | Pfam family (Pfam family ID)**	**GI | Pfam family (Pfam family ID)**			
Aap	254780173 | AapP	254780172 | BPD_transp_1 (CL0404) | BPD_transp_1 (PF00528)<br>254780171 | BPD_transp_1 (CL0404) | BPD_transp_1 (PF00528)	254780170 | SBP_bac_3 (PF00497)	general L-amino acid	importer	3.A.1.3.8
Pst	254780704 | PstB	255764486 | BPD_transp_1 (CL0404) | BPD_transp_1 (PF00528)<br>254780705 | BPD_transp_1 (CL0404) | BPD_transp_1 (PF00528)	254780707 | PBP_like_2 (PF12849)	Phosphate	importer	3.A.1.7.1
Cho	254780340 | ChoV	254780341 | BPD_transp_1 (CL0404) | BPD_transp_1 (PF00528)	254780342 | OpuAC (PF04069)	choline/acetylcholine (vitamin Bp)	importer	3.A.1.12.7
Nrt/Ssu/Tau-like	254780596 | NrtD/SsuB/TauB-like	255764497 (2×) | BPD_transp_1 (CL0404) | BPD_transp_1 (PF00528)	N/A	Oxoacid ions	importer	3.A.1.17
Thi	254780559 | ThiQ	255764501 (2×) | BPD_transp_1 (CL0404) | BPD_transp_1 (PF00528)	254780561 | SBP_bac_1 (PF01547)	Thiamine (vitamin B1)	importer	3.A.1.19.1
Znu	254780718 | ZnuC	254780719 | Membrane_trans (CL0142) | ABC-3 (PF00950)	254780717 | SBP_bac_9 (PF01297)	Zinc	importer	3.A.1.15.5
Sit	254780538 | SitB	254780539 | Membrane_trans (CL0142) | ABC-3 (PF00950)<br>254780540 | Membrane_trans (CL0142) | ABC-3 (PF00950)	254780537 | SBP_bac_9 (PF01297)	Manganese and iron	importer	3.A.1.15.9
Lin	254780139 | LinL	254780138 | DUF140 (PF02405)	254780140<br>254780141	Membrane lipids	importer*	3.A.1.27.1
Lol	254780871 | LolD	peg.788**, peg.789** | BPD_transp_1 (CL0404) | FtsX (PF02687)	254780798 | DUF330 (PF03886)	Lipoprotein	exporter*	3.A.1.125
Lpt	254780744 | LptB	255764468 | BPD_transp_1 (CL0404) | YjgP_YjgQ (PF03739)<br>255764469 | BPD_transp_1 (CL0404) | YjgP_YjgQ (PF03739)	254780745 | OstA (PF03968)<br>254780746<br>254780395 | OstA_C (PF04453)	Lipopolysaccharide	exporter*	1.B.42.1.2
Msb1	254780917_C | MsbA1	254780917_N | ABC_membrane (CL0241) | ABC_membrane (PF00664)	N/A	multidrug/lipid	exporter	3.A.1.106.1
Msb2	254780193_C | MsbA2	254780193_N | ABC_membrane (CL0241) | ABC_membrane (PF00664)	N/A	multidrug/lipid	exporter	3.A.1.106.1
Atm	254780576_C | AtmA	254780576_N | ABC_membrane (CL0241) | ABC_membrane (PF00664)	N/A	Heavy metal	exporter	3.A.1.106.1
Prt	255764467_C | PrtD	255764467_N | ABC_membrane (CL0241) | ABC_membrane (PF00664)	254780386<br>254780930 | OEP (PF02321)	Type I protein secretion	exporter	3.A.1.110.3
Suf	254781060 | SufC	N/A	N/A	Fe-S assembly	Non-transport	N/A
Uup	254781123 (2×) | Uup	N/A	N/A	Transposon excision regulation	Non-tranport	N/A
ChvD	254780273 (2×) | chvD	N/A	N/A	Virulence gene regulation	Non-tranport	N/A
UvrA	254780184(2×) | UvrA	N/A	N/A	DNA repair	Non-tranport	N/A
MutS	254780750 | MutS	N/A	N/A	DNA repair	Non-tranport	N/A

RecF	254780766 | RecF	N/A	N/A	DNA repair	Non-tranport	N/A
RecN	255764514 | RecN	N/A	N/A	DNA repair	Non-tranport	N/A
Rad50	254780640 | Rad50	N/A	N/A	DNA repair	Non-tranport	N/A
N/A	N/A	254781113 | BPD_transp_1 (CL0404) | BPD_transp_1 (PF00528)	254781112 | SBP_bac_3 (PF00497)	N/A	N/A	3.A.1.3
N/A	N/A	254780896 | membrane_trans (CL0142) | BPD_transp_2 (PF02653)	N/A	N/A	N/A	3.A.1.4.1
N/A	N/A	N/A	254780563***, 254780564***	N/A	N/A	N/A
