# Supplementary material for: The ABC transporters in Candidatus Liberibacter asiaticus
Source: Proteins. 2012 Jul 31;80(11):2614–28. doi: 10.1002/prot.24147 (PMC3688454; doi:10.1002/prot.24147)
Supplement: Supplementary file 11 [file prot0080-2614-sd11.pdf]

Table SII

gi	Seed ID	Seed annotation	NCBI annotation	KEGG pathway	COG classification
**254780173**	537021.9.peg.58	ABC transporter related protein	Amino acid ABC transporter, ATP-binding protein	ABC transporter related protein; K09972 general L-amino acid transport system ATP-binding protein [EC:3.6.3.-]	COG1126 ABC-type polar amino acid transport system, ATPase component
**254780172**	537021.9.peg.57	general L-amino acid transport system permease protein	Glutamate Aspartate transport system permease protein GltK (TC 3.A.1.3.4)	aapM; general L-amino acid transport system permease protein; K09971 general L-amino acid transport system permease protein	COG0765 ABC-type amino acid transport system, permease component
**254780171**	537021.9.peg.56	ABC transporter membrane spanning protein (amino acid)	Glutamate Aspartate transport system permease protein GltJ (TC 3.A.1.3.4)	aapQ; ABC transporter membrane spanning protein (amino acid); K09970 general L-amino acid transport system permease protein	COG4597 ABC-type amino acid transport system, permease component
**254780170**	537021.9.peg.55	cationic amino acid ABC transporter, periplasmic binding protein	Glutamate Aspartate periplasmic binding protein precursor GltI (TC 3.A.1.3.4)	cationic amino acid ABC transporter, periplasmic binding protein; K09969 general L-amino acid transport system substrate-binding protein	COG0834 ABC-type amino acid transport/signal transduction systems, periplasmic component/domain
**254780704**	537021.9.peg.538	ABC transporter, nucleotide binding/ATPase protein	Phosphate transport ATP-binding protein PstB (TC 3.A.1.7.1)	pstB; ABC transporter, nucleotide binding/ATPase protein; K02036 phosphate transport system ATP-binding protein [EC:3.6.3.27]	COG1117 ABC-type phosphate transport system, ATPase component
**255764486**	537021.9.peg.536	ABC transporter, membrane spanning protein	Phosphate transport system permease protein PstC (TC 3.A.1.7.1)	pstC; ABC transporter, membrane spanning protein; K02037 phosphate transport system permease protein	COG0573 ABC-type phosphate transport system, permease component
**254780705**	537021.9.peg.537	phosphate ABC transporter, permease protein PstA	Phosphate transport system permease protein PstA (TC 3.A.1.7.1)	pstA; phosphate ABC transporter, permease protein PstA; K02038 phosphate transport system permease protein	COG0581 ABC-type phosphate transport system, permease component
**254780707**	537021.9.peg.535	putative phosphate-binding periplasmic protein	Phosphate ABC transporter, periplasmic phosphate-binding protein PstS (TC 3.A.1.7.1)	putative phosphate-binding periplasmic protein; K02040 phosphate transport system substrate-binding protein	COG0226 ABC-type phosphate transport system, periplasmic component
**254780340**	537021.9.peg.239	proline/glycine betaine ABC transporter, ATP-binding protein	L-proline glycine betaine ABC transport system permease protein ProV (TC 3.A.1.12.1)	proline/glycine betaine ABC transporter, ATP-binding protein; K02000 glycine betaine/proline transport system ATP-binding protein [EC:3.6.3.32]	COG4175 ABC-type proline/glycine betaine transport system, ATPase component
**254780341**	537021.9.peg.240	proline/glycine betaine ABC transporter, permease protein	L-proline glycine betaine ABC transport system permease protein ProW (TC 3.A.1.12.1)	proline/glycine betaine ABC transporter, permease protein; K02001 glycine betaine/proline transport system permease protein	COG4176 ABC-type proline/glycine betaine transport system, permease component
**254780342**	537021.9.peg.241	substrate-binding region of ABC-type glycine betaine transport system	putative glycine betaine-binding ABC transporter protein	substrate-binding region of ABC-type glycine betaine transport system; K02002 glycine betaine/proline transport system substrate-binding protein	COG2113 ABC-type proline/glycine betaine transport systems, periplasmic components
**254780596**	537021.9.peg.648	ABC transporter nucleotide binding/ATPase protein	ABC-type nitrate/sulfonate/bicarbonate transport system, ATPase component	ABC transporter nucleotide binding/ATPase protein; K02049 sulfonate/nitrate/taurine transport system ATP-binding protein	COG1116 ABC-type nitrate/sulfonate/bicarbonate transport system, ATPase component
**255764497**	537021.9.peg.647	ABC transporter permease	ABC-type anion transport system, duplicated permease component	ABC transporter permease; K02050 sulfonate/nitrate/taurine transport system permease protein	COG4986 ABC-type anion transport system, duplicated permease component
**254780559**	537021.9.peg.686	thiamine transporter ATP-binding subunit	Thiamin ABC transporter, ATPase component / Thiamine transport ATP-binding protein thiQ	thiQ; thiamine transporter ATP-binding subunit; K02062 thiamine transport system ATP-binding protein	with TbpA and ThiP is part of the thiamine and TPP transport system

**255764501**	537021.9.peg.685	thiamine transporter membrane protein	Thiamin ABC transporter, transmembrane component	thiP; thiamine transporter membrane protein; K02063 thiamine transport system permease protein	permease; with TbpA and ThiQ functions in transport of thiamine and thiamine pyrophosphate into the cell; repressed in presence of exogenous thiamine
**254780561**	537021.9.peg.684	thiamine transporter substrate binding subunit	Thiamin ABC transporter, substrate-binding component	tbpA; thiamine transporter substrate binding subunit; K02064 thiamine transport system substrate-binding protein	COG4143 ABC-type thiamine transport system, periplasmic component
**254780718**	537021.9.peg.522	putative high-affinity zinc uptake system ATP-binding component of ABC transporter protein	Zinc ABC transporter, ATP-binding protein ZnuC	znuC; putative high-affinity zinc uptake system ATP-binding component of ABC transporter protein; K09817 zinc transport system ATP-binding protein [EC:3.6.3.-]	COG1121 ABC-type Mn/Zn transport systems, ATPase component
**254780719**	537021.9.peg.521	zinc uptake ABC transporter, permease protein	Zinc ABC transporter, inner membrane permease protein ZnuB	znuB; zinc uptake ABC transporter, permease protein; K09816 zinc transport system permease protein	COG1108 ABC-type Mn2+/Zn2+ transport systems, permease components
**254780717**	537021.9.peg.524	zinc uptake ABC transporter	Zinc ABC transporter, periplasmic-binding protein ZnuA	znuA; zinc uptake ABC transporter; K09815 zinc transport system substrate-binding protein	COG4531 ABC-type Zn2+ transport system, periplasmic component/surface adhesin
**254780538**	537021.9.peg.708	ABC transporter, nucleotide binding/ATPase protein (iron)	Manganese ABC transporter, ATP-binding protein SitB	ABC transporter, nucleotide binding/ATPase protein (iron); K11607 manganese/iron transport system ATP-binding protein	COG1121 ABC-type Mn/Zn transport systems, ATPase component
**254780539**	537021.9.peg.707	ABC transporter, membrane spanning protein (iron)	Manganese ABC transporter, inner membrane permease protein SitC	ABC transporter, membrane spanning protein (iron); K11605 manganese/iron transport system permease protein	COG1108 ABC-type Mn2+/Zn2+ transport systems, permease components
**254780540**	537021.9.peg.706	ABC transporter, membrane spanning protein (iron transport)	Manganese ABC transporter, inner membrane permease protein SitD	ABC transporter, membrane spanning protein (iron transport); K11606 manganese/iron transport system permease protein	COG1108 ABC-type Mn2+/Zn2+ transport systems, permease components
**254780537**	537021.9.peg.709	periplasmic solute binding protein	Manganese ABC transporter, periplasmic-binding protein SitA	periplasmic solute binding protein; K11604 manganese/iron transport system substrate-binding protein	COG0803 ABC-type metal ion transport system, periplasmic component/surface adhesin
**254780139**	537021.9.peg.18	putative ATP-binding component of ABC transporter	probable ABC transporter, ATP-binding protein	putative ATP-binding component of ABC transporter; K02065 putative ABC transport system ATP-binding protein	COG1127 ABC-type transport system involved in resistance to organic solvents, ATPase component
**254780138**	537021.9.peg.17	ABC transporter	ABC-type transport system involved in resistance to organic solvents, permease component USSDB6A	ABC transporter; K02066 putative ABC transport system permease protein	COG0767 ABC-type transport system involved in resistance to organic solvents, permease component
**254780140**	537021.9.peg.19	putative ABC transporter, substrate-binding protein	putative solute-binding component of ABC transporter	putative ABC transporter, substrate-binding protein; K02067 putative ABC transport system substrate-binding protein	COG1463 ABC-type transport system involved in resistance to organic solvents, periplasmic component
**254780917**	537021.9.peg.837	ABC transporter, nucleotide binding/ATPase protein	Y4gM	mdlB; ABC transporter, nucleotide binding/ATPase protein; K06147 ATP-binding cassette, subfamily B, bacterial	COG1132 ABC-type multidrug transport system, ATPase and permease components
**254780576**	537021.9.peg.668	ABC transporter related protein	putative ATP-binding component of ABC transporter	ABC transporter related protein; K06147 ATP-binding cassette, subfamily B, bacterial	COG5265 ABC-type transport system involved in Fe-S cluster assembly, permease and ATPase components
**254780193**	537021.9.peg.81	lipid A ABC exporter family, fused ATPase and inner membrane	hypothetical protein	lipid A ABC exporter family, fused ATPase and inner membrane subunits; K06147 ATP-binding cassette, subfamily B, bacterial	COG1132 ABC-type multidrug transport system, ATPase and permease components

		subunits			
**255764467**	537021.9.peg.287	Type I secretion system ATPase, PrtD	hypothetical protein	type I secretion system ATPase, PrtD; K06148 ATP-binding cassette, subfamily C, bacterial	COG4618 ABC-type protease/lipase transport system, ATPase and permease components
**254780871**	537021.9.peg.790	lipoprotein-releasing system ATP-binding protein lolD	Lipoprotein releasing system ATP-binding protein LolD	lipoprotein-releasing system ATP-binding protein LolD; K09810 lipoprotein-releasing system ATP-binding protein [EC:3.6.3.-]	COG1136 ABC-type antimicrobial peptide transport system, ATPase component
**N/A**	537021.9.peg.788	N/A	Lipoprotein releasing system transmembrane protein LolC		N/A
**N/A**	537021.9.peg.789	N/A	hypothetical protein		N/A
**254780744**	537021.9.peg.496	ABC transporter nucleotide binding/ATPase protein	Lipopolysaccharide ABC transporter, ATP-binding protein LptB	ABC transporter nucleotide binding/ATPase protein; K06861 lipopolysaccharide export system ATP-binding protein [EC:3.6.3.-]	COG1137 ABC-type (unclassified) transport system, ATPase component
**255764468**	537021.9.peg.295	permease protein	hypothetical protein	permease protein; K07091 lipopolysaccharide export system permease protein	COG0795 Predicted permeases
**255764469**	537021.9.peg.296	putative permease protein	putative permease protein	putative permease protein; K11720 lipopolysaccharide export system permease protein	COG0795 Predicted permeases
**254781060**	537021.9.peg.995	ABC transporter, nucleotide binding/ATPase protein	Iron-sulfur cluster assembly ATPase protein SufC	ABC transporter, nucleotide binding/ATPase protein; K09013 Fe-S cluster assembly ATP-binding protein	COG0396 ABC-type transport system involved in Fe-S cluster assembly, ATPase component
**254781123**	537021.9.peg.1057	putative ABC transporter, ATP-binding protein	ABC transporter, ATP-binding protein	putative ABC transporter, ATP-binding protein	COG0488 ATPase components of ABC transporters with duplicated ATPase domains
**254780273**	537021.9.peg.164	putative ABC transporter ATP-binding protein	ABC transporter, ATP-binding protein	putative ABC transporter ATP-binding protein	ChvD; in Agrobacterium tumefaciens, mutations in both Walker boxes were found to affect virulence
**254781112**	537021.9.peg.1046	putative amino acid-binding periplasmic ABC transporter protein	PUTATIVE AMINO ACID-BINDING PERIPLASMIC ABC TRANSPORTER PROTEIN	putative amino acid-binding periplasmic ABC transporter protein; K02030 polar amino acid transport system substrate-binding protein	COG0834 ABC-type amino acid transport/signal transduction systems, periplasmic component/domain
**254781113**	537021.9.peg.1047	amino acid ABC transporter (permease)	amino acid ABC transporter, permease protein	amino acid ABC transporter (permease); K02029 polar amino acid transport system permease protein	COG0765 ABC-type amino acid transport system, permease component
**254780896**	537021.9.peg.812	ABC transporter membrane spanning protein (branched chain amino acid)	Branched-chain amino acid transport system permease protein LivM (TC 3.A.1.4.1)	ABC transporter membrane spanning protein (branched chain amino acid); K01998 branched-chain amino acid transport system permease protein	COG4177 ABC-type branched-chain amino acid transport system, permease component
**254780564**	537021.9.peg.680	hypothetical protein	hypothetical protein	hypothetical protein	COG4143 ABC-type thiamine transport system, periplasmic component
**254780563**	537021.9.peg.681	extracellular solute-binding protein	iron ABC transporter, periplasmic iron-binding protein	extracellular solute-binding protein; K02012 iron(III) transport system substrate-binding protein	COG1840 ABC-type Fe3+ transport system, periplasmic component
**254780141**	537021.9.peg.20	putative ABC transporter protein	hypothetical protein	putative ABC transporter protein	COG3218 ABC-type uncharacterized transport system, auxiliary component

**254780798**	537021.9.peg.436	possible lolA type protein	outer membrane lipoprotein carrier protein LolA	possible LolA type protein	N/A
**254780745**	537021.9.peg.495	OstA family protein	hypothetical protein	OstA family protein; K09774 lipopolysaccharide export system protein LptA	COG1934 Uncharacterized protein conserved in bacteria
**254780746**	537021.9.peg.494	hypothetical protein	hypothetical protein	hypothetical protein; K11719 lipopolysaccharide export system protein LptC	
**254780395**	537021.9.peg.297	organic solvent tolerance protein	Outer membrane protein Imp, required for envelope biogenesis / Organic solvent tolerance protein precursor	organic solvent tolerance protein; K04744 LPS-assembly protein	COG1452 Organic solvent tolerance protein OstA
**254780386**	537021.9.peg.288	Type I secretion membrane fusion protein, HlyD	Alkaline protease secretion protein AprE	type I secretion membrane fusion protein, HlyD; K02022	COG0845 Membrane-fusion protein
**254780930**	537021.9.peg.850	nodulation protein (outer membrane efflux protein)	RND efflux system, outer membrane lipoprotein CmeC	nodT; nodulation protein (outer membrane efflux protein)	COG1538 Outer membrane protein
**254780184**	537021.9.peg.70	excinuclease ABC subunit A	Excinuclease ABC subunit A	uvrA; excinuclease ABC subunit A; K03701 excinuclease ABC subunit A	COG0178 Excinuclease ATPase subunit
**254780750**	537021.9.peg.489	DNA mismatch repair protein	DNA mismatch repair protein MutS	mutS; DNA mismatch repair protein; K03555 DNA mismatch repair protein MutS	COG0249 Mismatch repair ATPase (MutS family)
**254780766**	537021.9.peg.469	recombination protein F	DNA recombination and repair protein RecF	recF; recombination protein F; K03629 DNA replication and repair protein RecF	COG1195 Recombinational DNA repair ATPase (RecF pathway)
**255764514**	537021.9.peg.1111	DNA repair protein RecN	DNA repair protein RecN	recN; DNA repair protein RecN; K03631 DNA repair protein RecN (Recombination protein N)	COG0497 ATPase involved in DNA repair
**254780640**	N/A	hypothetical protein	N/A	hypothetical protein	COG1196 Chromosome segregation ATPases
