# Supplementary material for: The ABC transporters in Candidatus Liberibacter asiaticus
Source: Proteins. 2012 Jul 31;80(11):2614–28. doi: 10.1002/prot.24147 (PMC3688454; doi:10.1002/prot.24147)
Supplement: Supplementary file 9 [file prot0080-2614-sd9.pdf]

```
ALD_HUMA             GIICENIPIVTPEVVVASLNIRVEEGMHLLITGPNGCGKSSLFRILGGLWPTYGGVLYKPPPQRMFYIPQRPYMSVGSLRDQVIYPDSSEQDLEAILDVVHLHHILEAMKDVLSGGEKQRIGMARMFYHRPKYALLDECTSAVSIDVEGKIFQAAKDAGIALLSITHRPSLWKYHTHLLQFDEGGWKFE-KLDSAA
1_ABCC8_HUMAN        -VQIMGGYFTWTIPTLSNITIRIPRGQLTMIVGQVGCGKSSLLLAALGEMQKVSGAVFWSSGPVAYASQKPWLLNATVEENIIFESPNKQRYKMVIEACSLQPDIQTQGINLSGGQRQRISVARALYQHANVVFLDDPFSALDIHLSDHLMQAGLELKRTVVLVTHKLQYLPHADWIIAMKDGTIQREGTLKDFQ
HLYB_ECOLI           DITFRNIRFRYKPVILDNINLSIKQGEVIGIVGRSGSGKSTLTKLIQRFYIPENGQVLIDGRQVGVVLQDNVLLNRSIIDNISLANPSVEKVIYAAKLAGAHDFINTIGAGLSGGQRQRIAIARALVNNPKILIFDEATSALDYESEHVIMRNMHKIGRTVIIIAHRLSTVKNADRIIVMEKGKIVEQGKHKELL
Q54203_STRGA         RPEHADGPMGTLALVDETTGLTVTPGTFHVVVAPSAEGVRLADRL----GGYDEAPVRHDGRRILVVDNRPRLFRGPLRASLDPHVT-DTAITAALRAAAAEDIVDTDGRHFSGGQRH-LVLARALLADPDVLIVVDPTSAVDAHTEDLIGRRLRAAGRTTVLVSSSPLLLNQADRVSFLSGGRVAAAGTHRELL
MDR1_MOUSE           NVKFNGVQFNYPIPVLQGLSLEVKKGQTLALVGSSGCGKSTVVQLLERFYDPMAGSVFLDGAHLGIVSQEPILFDCSIAENIAYGDNSHEEIVRAAKEANIHQFINTRGTQLSGGQKQRIAIARALVRQPHILLLDEATSALDTESEKVVQEALDKAGRTCIVIAHRLSTIQNADLIVVIENGKVKEHGTHQQLL
LMRA_LACLA           TLSARHVDFAYDEQILRDISFEAQPNSIIAFAGPSGGGKSTIFSLLERFYQPTAGEITIDGSQIGFVSQDSAIMAGTIRENLTYGLETDEDLWQVLDLAFARSFVNTEGVKISGGQRQRLAIARAFLRNPKILMLDEATASLDSESESMVQKALDSLGRTTLVIAHRLSTIVDADKIYFIEKGQITGSGKHNELV
ATM1_YEAST           DITFENVTFGYHRKILKNASFTIPAGWKTAIVGSSGSGKSTILKLVFRFYDPESGRILINGKVIGVVPQDTPLFNDTIWENVKFGRITDEEVITVVEKAQLAPLIDTIGLMISGGEKQRLAIARVLLKNARIMFFDEATSALDTHTEQALLRTIDNFSRTSVYIAHRLRTIADADKIIVLDNGRVREEGKHLELL
MSBA_ECOLI           DVEFRNVTFTYPVPALRNINLKIPAGKTVALVGRSGSGKSTIASLITRFYDIDEGEILMDGNQVALVSQNVHLFNDTVANNIAYARTSREQIEEAARMAYAMDFIDTVGVLLSGGQRQRIAIARALLRDSPILILDEATSALDTESERAIQAALDELNRTSLVIAHRLSTIEKADEIVVVEDGVIVERGTHNDLL
Q9R7V3_YERPE         SITFEQVSFHYPGAALQEVSFHVPAGQIVALVGPSGAGKSTVARLLLRYADPDKGHIRIGGKQLSFVFQDNFLFADTIANNIRLGAPPLEAVIAAARVAQAHDFINTRGVFLSGGQRQRITIARALLQDRPILVLDEATAFADPENEAALIKALAAAGRTVIMVAHRLSMVTQADVILLFSDGQLREMGNHTQLL
MDL1_YEAST           PIVFKNVSFTYPHQIFKDLNITIKPGEHVCAVGPSGSGKSTIASLLLRYYDVNSGSIEFGDRLIGYVQQEPLLFNGTILDNILYCIPQDDRIRRAIGKANCTKFLQTMGAQLSGGQKQRIALARAFLLDPAVLILDEATSALDSQSEEIVAKNLQRRGFTTISIAHRLSTIKHSTRVIVLGHGSVVETGSFRDLI
MESD_LEUME           SLVADHITYKYGAPAIDDVSLTITAGEKIALVGISGSGKSTLVKLLVNFFQPESGTISLGPGHINYLPQEPFIFSGSIMENLLLGAK--EDIIRAVEIAEIKDDIGTESGNISGGQKQRIALARAILVDSPVLILDESTSNLDVLTEKKIIDNLMKLEKTIIFVAHRLTISQRVDRILTMQSGKIIEDGTHDTLL
CYDC_ECOLI           SLTLRDVQFTYPQQALKGISLQVNAGEHIAILGRTGCGKSTLLQQLTRAWDPQQGEILLNDQTISVVPQRVHLFSATLRDNLLLASPSDEALSEILRRVGLEKLLNSWGRQLSGGELRRLAIARALLHDAPLVLLDEPTEGLDATTESQILELLAEMEKTVLMVTHRLRGLSRFQQIIVMDNGQIIEQGTHAELL
NIST_LACLA           TVKVINLSYVYPAFALKNINLSFEKGELTAIVGKNGSGKSTLVKIISGLYQPTMGIIQYDKKNISVLFQDFVKYELTIRENIGLSD-EDEKIIKVLDNLGLDFLKDTQGHQLSGGQWQKIALARTFFKKASIYILDEPSAALDPVAEKEIFDYFVALNNISIFISHSLNAARKANKIVVMKDGQVEDVGSHDVLL
TAP1_HUMAN           LVQFQDVSFAYPVLVLQGLTFTLRPGEVTALVGPNGSGKSTVAALLQNLYQPTGGQLLLDGRQVAAVGQEPQVFGRSLQENIAYGL-TMEEITAAAVKSGAHSFIDTEGSQLSGGQRQAVALARALIRKPCVLILDDATSALDANSQLQVEQLLYESSRSVLLITQHLSLVEQADHILFLEGGAIREGGTHQQLM
MRP1_HUMAN           RVEFRNYCLRYRDFVLRHINVTINGGEKVGIVGRTGAGKSSLTLGLFRINESAEGEIIIDGFKITIIPQDPVLFSGSLRMNLDPFSQSDEEVWTSLELAHLKDFVDHEGENLSVGQRQLVCLARALLRKTKILVLDEATAAVDLETDDLIQSTIRTQDCTVLTIAHRLNTIMDYTRVIVLDKGEIQEYGAPSDLL
CFTR_HUMAN           QMTVKDLTAKYTNAILENISFSISPGQRVGLLGRTGSGKSTLLSAFLRLL-NTEGEIQIDGKAFGVIPQKVFIFSGTFRKNLDPYEQSDQEIWKVADEVGLRSVIDFVGCVLSHGHKQLMCLARSVLSKAKILLLDEPSAHLDPVTYQIIRRTLKQADCTVILCEHRIEAMLECQQFLVIEENKVRQYDSIQKLL
2_ABCC8_HUMAN        KIQIQNLSVRYDKPVLKHVNALISPGQKIGICGRTGSGKSSFSLAFFRMVDTFEGHIIIDGSRLSIILQDPVLFSGTIRFNLDPERKSDSTLWEALEIAQLKLVVDAIGENFSQGQRQLFCLARAFVRKTSIFIMDEATASIDMATENILQKVVMTADRTVVTIAHRVHTILSADLVIVLKRGAILEFDKPEKLL
MsbA2_254780193      SVSFRSVSFVYPRSILKNVNFTVQPGETIALVGDSGAGKTSIFSLILRFYDPCSGSVEIEGRFIAWVPQHPIMIAASVYDNIAIGCPTRKEIQNAAMLAQAHEFIDTIAVHLSTGQIQRIAIARAILKDSPILLLDEMGSALDIENEQKIWKVLREQGRTTIFASHRLSAIQDADMVFVLHDQVIVEKGVHEELL
AtmA_254780576       GHIVFNNVSFSYNCIFKGISFEIAPSKKTALIGESGVGKSTVAKLLYRLYDIKGGIITIDGQTIGIIPQDTILFNDTLRYNILYGPNSDQELCAAVEVAQLKSFIDTVGLKLSGGEKQRVSIARAILKNPPIIIFDEATSSLDIITERHIQTALATLNRTTLVIAHRLSTITDADNIIVFDQGTVVESGSHENLI
AtmA_94309336        GAEVRFRHVGFGRVILDDVDFTIAAGTTTAVVGHSGSGKSTLARLLFRFYDVTRGSIEVDGASIGIVPQDTVLFNDSIYYNIAYGRPTREEVIEAARAAQIDTFIDTPGLKLSGGEKQRVAIARTLLKNPPILVFDEATSALDSRTEQAIQSELMRLNRTTLLIAHRLSTVVHADQILVMDRGRIIERGTHAELM
MsbA1_254780917      TTVFRDVFFSYKHPVLSGINLCFKSGKMTALVGPSGSGKSTIINLLMRMYDPSSGSIEIDGERISYVGQDVFLFSNTVRYNILIGRPTEEEMIEIAKSANAHDFIDTYGSNLSGGQKQRIAIARAMLRDGHILVLDEATSALDTHTENLVRQALSRLGRTTIVIAHRISTIIEADHIIFIEDGKVSESGDQKYLL
PRTD_ERWCH           HLSVEQVSLRTANTRLQNIHFSLQAGETLVILGASGSGKSSLARLLVGAQSPTQGKVRLDGPTIGYLPQDVQLFKGSLAENIARFGDDPEKVVAAAKLAGVHELIDTEGGGLSGGQRQRIGLARAMYGDPCLLILDEPNASLDSEGDQALMQAIVALGATVVLITHRPALTTLAQKILILHEGQQQRMGLARDVL
PrtD_255764467       TITAKNIVFRDKRLICQNLSFTIPEGSCCIIAGPSGCGKSNFLLCMLGLLTLEKGTISFGDAQIGYLSQHCSLFPISITENIILSH-SLHIAQKTAKIVGCHEDIQTTSDEIPYNIVQKIRLSRIIANNPAILLMDEPLYHLDNEAKNNFYTLLKQFKKTIVIISHDPTIISMSDFSLIFHPIQGSLFGLTQDIF
PrsD_195970027       RLTVEGLASGPPRLVVSDVNFTVRAGGAVGVIGPSASGKSSLARAILGIWPAYRGSVRLDGKHVGYLPQDVELFAGTIAQNICRFA-TSEAIVAAAKAARVNDLIDTEGMTLSAGQRQRLA--RALYGDPFLVVLDEPNSNLDAEGEQALSEAIMSVGGIVIVVAHRPSALASVDLVLMMNEGRMQAFGPKEQVL
CBIO_SALTY           MLATSDLWFRYQEPVLKGLNMDFSLSPVTGLVGANGCGKSTLFMNLSGLLRPQKGAVLWQGQQVATVFQDPQIFYTDIDSDIAFSLRITRRVDEALTLVDAQHFRHQPIQCLSHGQKKRVAIAGALVLQARYLLLDEPTAGLDPAGRTQMIAIIRRIGNHVIISSHDIDLIYISDAVYVLRQGQILTHGAPGEVF
Q6GUB2_RHIET_biotin  NIQFESAGVSFGRVALEPLTLAIT-GKRIGVIGLNGSGKTTFARLVNGLTKPTTGRVIVNGGFIFQSPQNQ-IILPIVKYDIAFGLKIEARVEGVLARFGAEALADRRAHELSGGELQVAALCSVLATGPGILILDEPTNQLDLKNRALVERIIAGLPESAIVITHDLELIAGFERVLVFHEGRLAADEPAAEAI
Q9Z402_PSEPUos       AVELKGVTFKRGSRIFSNVDIRIPRGKVTGIMGPSGCGKTTLLRLMGAQLRPSSGEVWVAGKQMGVLFQSGALFDLDVFENVAFPLRIRDIVLMKLQAVGLRGAIDLMPDELSGGMKRRVALARAIALDPQILMYDEPFVGQDPIAMGVLVRLIRLLGITSIVVSHDLAETAIADYIYVVGDGQVLGQGTPDELM
LinL_139001579       IISVRNLRNSFGQVVHEGLNLDVRKGEILGVVGGSGTGKSVLMRSIIGLQTPDEGEIHVFGKRWGVLFQGGALFTLTVAENVEVPIRRDEIAAYKIRLTGLPAEAPKYPAELSGGMKKRAGLARALALDPDLLFLDEPTAGLDPIGAAAFDDQTRRLGLTVFLITHDLDTLYICDRVAVLADRKVTAVGTIDELL
LinL_254780139       ILSVRNLTIELNRIILNNINLDILRGEILGVIGSSGTGKSILMRGILGLIPRVSGEVKVLGAKLGVLFQHGALFSMTVQENISMPIKIEHIVHTKIAIVGLPPETNLMPFQLSGGMIKRVSLARALAIDPDLLFLDEPTSGLDPIAAEEFDKLLIKLGLSVYMITHDLNNLITCNRVAVIQKKGIMKEGTISDIV
LOLD_ECOLI           LLQCDNLCKRYQTDVLHNVSFSVGEGEMMAIVGSSGSGKSTLLHLLGGLDTPTSGDVIFNGQKLGFIYQFHHLLDFTALENVAMPLLINSRALEMLKAVGLDHRANHRPSELSGGERQRVAIARALVNNPRLVLADEPTGNLDARNADSIFQLLGELGTAFLVVTHDLQLAKRMSRQLEMRDGRLTAELSLMGAE
LolD_254780871       VLLLQNVKHSYRFPVLENVHLSLKKGEIVALVSPSGTGKSTILHIAGLLEVPDQGNVIIANSKIGFVYQEHRLLDFSVIENIIFPQIAYQRAMDLLSYMDMSQYANRRSSDISGGEQQRVAICRAIANKPLIILADEPTGNLDLKTAQQVFSILKYLGLAALIATHNHDLASQMDRQITIRDGMIADL-------
FTSE_ECOLI           MIRFEHVSKAYLRQALQGVTFHMQPGEMAFLTGHSGAGKSTLLKLICGIERPSAGKIWFSGRQIGMIFQDHHLLDRTVYDNVAIPLIIRRRVSAALDKVGLLDKAKNFPIQLSGGEQQRVGIARAVVNKPAVLLADEPTGNLDDALSEGILRLFEEFGVTVLMATHDINLISRSYRMLTLSDGHLHGGVGHE---
LptB_254780744       LLSACSLTKKYNVYVVKGVDLYVKPGEIVGLLGPNGAGKTTFFYMMTGLICADSGSIKIDGLGISYLPQEASIFGLTVEENILAVLERHEKLDSLLKEFNITKWRHVLAPLLSGGQRRRLEIARSLVSFPSYILLDEPFAGVDPVAISDIHVLIRDLGIGILITDHNVHEILLIDRAYIIHAGGVLAHGTVQEII
LPTB_ECOLI           TLTAKNLAKAYKRRVVEDVSLTVNSGEIVGLLGPNGAGKTTTFYMVVGIVPRDAGNIIIDDRGIGYLPQEASIFRLSVYDNLMAVLQREDRANELMEEFHIEHLRDSMGQSLSGGERRRVEIARALAANPKFILLDEPFAGVDPISVIDIKRIIEHLGLGVLITDHNVRETLVCERAYIVSQGHLIAHGTPTEIL
FHUC_ECOLI           TFALRNISFRVPRTLLHPLSLTFPAGKVTGLIGHNGSGKSTLLKMLGRHQPPSEGEILLDARKVAYLPQQLPPAGMTVRELVAIGRYDREKVEEAISLVGLKPLAHRLVDSLSGGERQRAWIAMLVAQDSRCLLLDEPTSALDIAHQVDVLSLVHRLGLTVIAVLHDINMAAYCDYLVALRGGEMIAQGTPAEIM
SitB_254780538       GLELKDISVTYPYCALEDVNLFIPENTITALIGLNGAGKSTLFQAIMGLIPLDKGSISIFNDLIAYIPQTEWTFPILVEDVVMMGRYDYRIVTEALENVGMSSMRQRQIGELSIGQQKRVFLARALAQKSQVIIMDEPFAAIDFKTEREMIFLLKSLDIMILVSTHNINSIPFCDRTIFLK-KTVLASGLTSDIF
SitB_16766168        AITVDQVTVTYRHTALRDATFQVPGGSIAALVGVNGSGKSTLFKALMGFVHLAQGDITILQNLIAYVPQSEWSFPVLVEDVVMMGRYDHACVDAALARVDMQEYRHRQIGELSGGQKKRVFLARAIAQDGQVILLDEPFTGVDVKTEARIIDLLRELGRTMLVSTHNLGSVTFCDYTVMIK-GTVLASGPTETTF
ZnuC_254780718       LISLSNTSFHKNYKILQDINFTIKPNEIVTLIGPNGSGKSTIAKLITGIIKPTIGSVKRHPLIVGYVPQKVNTLPLSLMRFMTLSMPSRDDVLQILDRVNLIGKYNRNIKDLSGGEFQRALLAKALLRKPNLLVLDEPLQGIDFPGELSLYELITSVGCGILLISHNLHMVMSTDTVICLN-NRICYQGPPQTIK
ZNUC_ECOLI           LVSLENVSVSFGRRVLSDVSLELKPGKILTLLGPNGAGKSTLVRVVLGLVTPDEGVIKRNGLRIGYVPQKLTTLPLTVNRFLRLRPGHKEDILPALKRVQAGHLINAPMQKLSGGETQRVLLARALLNRPQLLVLDEPTQGVDVNGQVALYDLIDQLDCGVLMVSHDLHLVMKTDEVLCLN-HHICCSGTPEVVS
LIVG_ECOLI           LLSVNGLMMRFGLLAVNNVNLELYPQEIVSLIGPNGAGKTTVFNCLTGFYKPTGGTILLRDMGVVRTFQHVRLFREMTVIENLLVAQHALDRAATWLERIGLLEHANRQASNLAYGDQRRLEIARCMVTQPEILMLDEPAAGLNPKETKELDELIAELNTTILLIEHDMKLVMISDRIYVVNQGTPLANGTPEQIR
AapP_254780173       AVEIKFLNKWYGFHALRDINLNVLRGERIVIIAGPSGSGKSTLIRCINHIEKHQAGEIIINGRDVGMVFQHFNLFNLSVLENCTLGPIAEELAMHYLNKVKIADKSHRSSSLLSGGESQRAAIARSLCMQPKIMLFDEPTSALDPEMVKEVLDIMIELGMTMVCVTHEMGFARVADRVVFMDQGQIVEQNSPEEFF
AapP_116251957       AVEIVNMNKWYGFHVLRDINLKVMRGERIVIAGPSGSGKSTMIRCINRLEEHQKGKIVVDGREVGMVFQHFNLFHLTILENCTLAPIAEEVAMHFLKRVKIPEQANKYPGQLSGGQQQRVAIARSLCMNPKIMLFDEPTSALDPEMIKEVLDTMVGLGMTMLCVTHEMGFARVANRVIFMDQGQIVEQNEPAAFF
ThiQ_254780559       MIKLDHLIYCYD-NLKMQFDFQVNKAERIVILGPSGAGKSTLLSLMAGFKYPTRGSIFLNGRPLSILFQENNLFHLTVWQNIALGIAQHAQVKQMLEKVFLEDFFNRFPSQMSGGQRQRVALARCLIRQKPILLLDEPFAVFDPALRHEILGLLKQVQLTLLMISHNLEDSMIATRFIVIAGGQIVYDGDPDRLM
ThiQ_16763496        MLKLIDITWLYH-HLPMRFTLAVERGEQVAILGPSGAGKSTLLNLIAGFLAPASGTLLIAGRPVSMLFQENNLFHLNVQQNIGLGLNQRREKRDAIAHQMGIESLMTRLPGELSGGQRQRVALARCLVREQPVLLLDEPFSALDPALRQEMLTLVSDIQLTLLMVSHSVEDAAIAPRSIVVADGRIAWQGKTDELL
Nlik_254780596       LLEIQDICQSFSLLVLDKVNFNVSSGEIVGLLGRSGSGKSTLLRIIAGLVSPSSGQVLFKGKDISVVFQTFALFWLTVLENVELGLKRRKRALSAIDLIGLDGFESAHTTEISGGMLQRVGFARAFVVFPSLLLMDEPFSALDVLTAENLRTDLIDLIESMLMVTHNIEEAVMCDRVLVFSPGRIAEEVLISIPH
NrtD_81300300        FLIVENVSKIYEYTVLDGVNLTVQEGEFICVIGHSGCGKSTLLNMVSGFNQPSHGSVRLKGPDRMVVFQNYALLWMTAYENVYLAVDKREIVREHLAMVGLTEAAEKKITQISGGMKQRVAIARALSIRPEVLILDEPFGALDAITKEELQEELLKIRCTVLMITHDIDEALLADRLVMMTNGPAANGEIMTIPF
SsuB_255767205       AVTISIKEKAFVNTVLENIELSIAPGEFLTLIGPSGCGKSTLLKIIAGLDSEYDGSVEINGIQQGFIFQEHRLFWLTVEQNIAADLNVKQKVDELIEIVRLKGSEKAYPRELSGGMSQRVAIARALLREPEVLLLDEPFGALDAFTRKHLQDVLLDIKTTMILVTHDIDESVLGNELAILKPGKIHKLMPIHLAY
TAUB_ECOLI           MLQISHLYADYGKPALEDINLTLESGELLVVLGPSGCGKTTLLNLIAGFVPYQHGSIQLAGAERGVVFQNEGLLWRNVQDNVAFGLQLRLEIAHQMLKKVGLEGAEKRYIWQLSGGQRQRVGIARALAANPQLLLLDEPFGALDAFTRDQMQTLLLKLGKQVLLITHDIEEAVMATELVLLSPGRVLERLP-----
ChoV_254780340       AVKFRDVDIVFGVSAVIKANLEVKKGEILVLMGLSGAGKSTLLRSINGLAPVVRGEVLVDTHTVSMVFQQFALLWRTVARNVELGLERKSRVAEHLEIVNLTKWADCKINMLSGGMKQRVGFARAFATGAPILLMDEPFSSLDPLIRMRLQDELLALKKTIVFVSHDINEAFLGNRIAIMEGGRIIQCGTPQEII
ChoV_15966154        AVVFKNVDIIFGVLGVAGASLTINEGEILVLMGLSGSGKSTLLRAVNGLAPVVRGEVEVKTHTVSMVFQQFALLWRTVADNVGFGLERRKRVGEQLELVNLAKWADRKVNELSGGMQQRVGLARAFATGAPILLMDEPFSALDPLIRTRLQDELLEFKKTIIFVSHDLDEAFIGNRIAIMEGGRIIQCGTPQEIV
PstB_254780704       KMISNKVSVCYGKRALFDINLKIPQNSVTALIGPSGCGKSTFLRCLNRMNCVFTGEIILDGARVGMVFQRPNPFPKSIYDNVAYGPRIDQIVEMSLKKASLFNEVDRLASLLSGGQQQRVCIARALAVNPEVILMDEPCSALDPIATASVEELIHELSLTIVIVTHSMQQAAVSQRAAMFHMGHLVEEDDTDKMF
PstB_16131593        KIQVRNLNFYYGFHALKNINLDIAKNQVTAFIGPSGCGKSTLLRTFNKMEQRAEGEILLDGAKVGMVFQKPTPFPMSIYDNIAFGVRMDERVQWALTKAALWNETDKLGYSLSGGQQQRLCIARGIAIRPEVLLLDEPCSALDPISTGRIEELITELDYTVVIVTHNMQQAACSDHTAFMYLGELIEFSNTDDLF
METN_ECOLI           MIKLSNITKVFHIQALNNVSLHVPAGQIYGVIGASGAGKSTLIRCVNLLERPTEGSVLVDGRQIGMIFQHFNLLSRTVFGNVALPLEVKRRVTELLSLVGLGDKHDSYPSNLSGGQKQRVAIARALASNPKVLLCDEATSALDPATTRSILELLKDIGLTILLITHEMDVVKICDCVAVISNGELIEQDTVSEVF
PHNC_ECOLI           IIRVEKLAKTFNHQALHAVDLNIHHGEMVALLGPSGSGKSTLLRHLSGLGDKSVGSHIELLAHTGYIFQQFNLVRLSVLENVLIGALQKQRALQALTRVGMVHFAHQRVSTLSGGQQQRVAIARALMQQAKVILADEPIASLDPESARIVMDTLRDIGITVVVTLHQVDYALYCERIVALRQGHVFYDGSSQQFD
HISP_SALTY           KLHVIDLHKRYGHEVLKGVSLQARAGDVISIIGSSGSGKSTFLRCINFLEKPSEGAIIVNGTRLTMVFQHFNLWHMTVLENVMEAPIARERALKYLAKVGIDERAGKYPVHLSGGQQQRVSIARALAMEPDVLLFDEPTSALDPELVGEVLRIMQQLGKTMVVVTHEMGFARVSSHVIFLHQGKIEEEGDPEQVF
OPPD_SALTY           LLEVNDLRVTFAVTAVNDLNFTLRAGETLGIVGESGSGKSQTAFALMGLATNIGGSATFNGEQISMIFQDPSLNYMRVGEQLMEVLMAFEESVRMLDAVKMPEARKRMPHEFSGGMRQRVMIAMALLCRPKLLIADEPTTALDVTVQAQIMTLLNELNTAIIMITHDLGVVAICDKVLVMYAGRTMEYGKARDVF
RBSA_ECOLI           LLQLKGIDKAFPVKALSGAALNVYPGRVMALVGENGAGKSTMMKVLTGIYTRDAGTLLWLGAGIGIIHQELNLIQLTIAENIFLGREMYAEADKLLAKLNLRFKSDKLVGDLSIGDQQMVEIAKVLSFESKVIIMDEPTDALTDTETESLFRVIRELGRGIVYISHRMKEIFICDDVTVFRDGQFIAEREVASLT
POTA_ECOLI           LVQLAGIRKCFDKEVIPQLDLTINNGEFLTLLGPSGCGKTTVLRLIAGLETVDSGRIMLDNRYVNTVFQSYALFHMTVFENVAFGLRITPRVMEALRMVQLETFAQRKPHQLSGGQQQRVAIARAVVNKPRLLLLDESLSALDYKLRKQMQNELKALGITFVFVTHDQEEALMSDRIVVMRDGRIEQDGTPREIY
DRRA_STRPE           AIETSGLVKVYNTRAVDGLDLNVPAGLVYGILGPNGAGKSTTIRMLATLLRPDGGTARVFGRISVTGQYASVDGLTGTENLVMMGRARERAAELIDGFGLGDARDRLLKTYSGGMRRRLDIAASIVVTPDLLFLDEPTTGLDPRSRNQVWDIVRALGTTVLLTTQYLDEADLADRIAVIDHGRVIAEGTTGELK
1_ABC1_HUMAN         -VSIQNLVKVYRKVAVDGLALNFYEGQITSFLGHNGAGKTTTMSILTGLFPPTSGTAYILGQNLGVCPQHNVLFMLTVEEHIWFYARVKAEMEQMALDVGLPSSKLKSTSQLSGGMQRKLSVALAFVGGSKVVILDEPTAGVDPYSRRGIWELLLKYGRTIILSTHHMDEADLGDRIAIISHGKLCCVGSSLFLK
2_ABC1_HUMAN         -LEIKELTKIYRKKPAVDRICVGIPPGECFGLLGVNGAGKSSTFKMLTGDTTVTRGDAFLNKQNMGYCPQFDAITLLTGREHVEFFALVGKVGEWAIRKLGLVKYGEKYAGNYSGGNKRKLSTAMALIGGPPVVFLDEPTTGMDPKARRFLWNCALSVGRSVVLTSHSMEECELCTRMAIMVNGRFRCLGSVQHLK
NOSF_PSEST           AVEIQGVSQRYGMTVLHDLNLNLGEGEVLGLFGHNGAGKTTSMKLILGLLSPSEGQVKVLGRQLGYLPENVTFYQLSGRETLRHFARALTQVDELLEQVGLAHAADRRVKTYSKGMRQRLGLAQALLGEPRLLLLDEPTVGLDPIATQDLYLLIDRLGTSIILCSHVLPGVEHINRAAILAKGCLQAVGSLSQLR
Q45404_BACSU         LLETKSLTKKFGETAVADVSLTVQKNSIYGLLGPNGAGKSTTLKILTGILRKTSGEILFDGQTIGSLIEAPPLYNLTAFENVKVHATSEDRIHSVLKTVDMQHAGKKRAGQFSMGMRQRLGIAIALLNHPKLLILDEPTNGLDPIGIQELRDLIRSFGITVILSSHILSEVEIADHIGIIDHGHLGYQGEINKAD
BCRA_BACLI           IIKTTDLTKMYGQKSVDHLNINVKQGDIYGFLGRNGAGKTTTIRMLLGLIKPTSGQIEIFGRRIGSIVEVPGFYNLTARENLLINAKKKNAIDEVLEIVGLQHETKKLVKGFKSLGMKQRLGIARALLHYPELLILDEPTNGLDPIGIKEMRRLIHSLNITIFISSHILSEIELVDHVGIIHEGKLLEEIPFDHLK
YHIH_ECOLI           AIEARDLTMRFGFVAVDHVNFRIPRGEIFGFLGSNGCGKSTTMKMLTGLLPASEGEAWLFGRRVGYMSQAFSLYELTVRQNLELHARIPARVAEMSERFKLNDVEDILPESLPLGIRQRLSLAVAVIHRPEMLILDEPTSGVDPVARDMFWQLMVDLKVTIFISTHFMNEAERCDRISLMHAGKVLASGTPQELV
KPST1_ECOLX          MIKIENLTKSYRHYVFKNLNIIFPKGYNIALIGQNGAGKSTLLRIIGGIDRPDSGNIITEHVGLAGGFQG----SLTGRENVKFVARLNERVDFVEEFSELGKYFDMPIKTYSSGMRSRLAFGLSMAFKFDYYLIDEITAVGDAKFKKKCSDIFDKIKSHLIMVSHSERALKYCDVAIYLNEGQGKFYKNVTEAI
CCMA_ECOLI           MLEARELLCERDRTLFSGLSFTLNAGEWVQITGSNGAGKTTLLRLLTGLSRPDAGEVLWQGQNLLWIGHQPGIKRLTALENLHFYH-DTAQCLEALAQAGLAGFEDIPVNQLSAGQQRRVALARLWLTRATLWILDEPFTAIDVNGVDRLTQRMAQHGGIVILTTHQPLNVASIRRISLTQ-TRAA---------
PDR5_YEAST           IFHWRNLCYEVQRRILNNVDGWVKPGTLTALMGASGAGKTTLLDCLAERTMGITGDILVNGRSIGYCQQQDLHLTATVRESLRFSAYKNRYVEEVIKILEMEKYADAVGEGLNVEQRKRLTIGVELTAKPKLLVLDEPTSGLDSQTAWSICQLMKKLGQAILCTIHQPSAILQFDRLLFMQGGKTVYFGDLGEGC
WHITE_DROME          GLFCNERHIPAPKHLLKNVCGVAYPGELLAVMGSSGAGKTTLLNALAFR-SPQGIQVSPSGARCAYVQQDDLFISLTAREHLIFQAMRVARVDQVIQELSLSKCQHTIVKGLSGGERKRLAFASEALTDPPLLICDEPTSGLDSFTAHSVVQVLKKLGKTVILTIHQPSSELFELFDKILLMAEGRVAFLGTPSEAV
SufC_254781060       MLEIKNLRAKIVTEIIRGLNLKVEPSEVVAIMGPNGSGKSTLSYLLSGHYEITAGDILYKGKGIFLAFQYPVEIGIIMMQFLKIALNFTRKVRKITAELGINMDMKRPNLGFSGGEKKIAEILQMRLLEPSLCILDETDSGLDIDALKIAAQGVNALDRSFLIITHYQRLLNIPDTIHVLHNGKIIKTGDYSIAL
SUFC_ECOLI           MLSIKDLHVSV-KAILRGLSLDVHPGEVHAIMGPNGSGKSTLSATLAGRYEVTGGTVEFKGEGIFMAFQYPVEIGVSNQFFLQTALNFQDLMEEKIALLKMPEDLTRSNVGFSGGEKKRNDILQMAVLEPELCILDESDSGLDIDALKVVADGVNSLKRSFIIVTHYQRILDIPDYVHVLYQGRIVKSGDFTLVK
1_ABCE1_HUMAN_1YQT   -NLEKETTHRYCAFKLHRLPI-PRPGEVLGLVGTNGIGKSTALKILAGKTYFRGSELQNYFLKAIIKPQYVPKAKGTVGSILDRK--ETKTQAIVCQQLDLTHLKERNVEDLSGGELQRFACAVVCIQKADIFMFDEPSSYLDVKQRLKAAITIRSLDRYIIVVEHDLSVLDLSDFICCLY-GVPSAYGVVTMPF
2_ABCE1_HUMAN        MYKYPGMKKKMGEFELAIVAGEFTDSEIMVMLGENGTGKTTFIRMLAGRKPDEGGEVPVL--NVSYKPQKISPKTGSVRQLLHEKIRHPQFVTDVMKPLQIENIIDQEVQTLSGGELQRVALALCLGKPADVYLIDEPSAYLDSEQRLMAARVVKRFKKTAFVVEHDFIMATLADRVIVFDPSKNTVANSPQTLL
1_UVRA_254780184     NISIRGAREH----NLQNISIELPRNKLIVMTGVSGSGKSSLAFDTIHAKPDVEQIDGLS--PTISIEQKNSHNRSTVGTITEIHDY--QKRIHFLVEIGLDYLTSRNSNMLSNGESQRIRLASQIGSAGVLYILDEPSIGLHQRDNTKLINTLKHLGNTVIVVEHDEETMLAADHIVDIGGGKIVAEGSPSQII
2_UVRA_254780184     MIHVINARSN----NLKNVTGSIPLGLFTAITGISGGGKSTFLISTLYKPGIYDRIDGLEYDKVVSINQSPGHTRSNPATYVGAFTP--HNKLTTLKEVGLGYIKGQSANTLSGGESQRVKLAKELSKQNTLYILDEPTTGLHYHDIAKLLNILHTLGNSIIVIEHNLEVIKTADWILDFGGGEIIASGPPEAIA
1_UVRA_ECOLI_2R6F    KIEVRGARTH----NLKNINLVIPRDKLIVVTGLSGSGKSSLAFDTLYYFPDVDHIEGLS--PAISIEQKSSHNRSTVGTITEIHDYEIGDRLKFLVNVGLNYLTSRSAETLSGGEAQRIRLASQIGAGGVMYVLDEPSIGLHQRDNERLLGTLIHLGNTVIVVEHDEDAIRAADHVIDIGGGEAQRVKLARELS
2_UVRA_ECOLI         VLKLTGARGN----NLKDVTLTLPVGLFTCITGVSGSGKSTLINDTLFEPAPYRDIQGLEHDKVIDIDQSPGRTRSNPATYTGVIEEALARKLQTLMDVGLTYIRGQSATTLSGGEAQRVKLARELSKRQTLYILDEPTTGLHFADIQQLLDVLHKLGNTIVVIEHNLDVIKTADWIVDLGGGEILVSGTPETVA
1_MSRA_STAEP         TIKFNQINHKLTLRSLNIDHLYAYQFEKIALIGGNGTGKTTLLNMIAQKTKPESGTVETN-GEIQYFEQLN----------------VENDFNTLDGSLMSELHIMHTTDSMSGGEKAKYKLANVISNYSPILLLDEPTNHLDKIGKDYLNNILKYYYGTLIIVSHDRALIDIADTIWDIQDGTIRVKGNYTQYQ
2_MSRA_STAEP         -IIAQNLTLVKGQKLLTQVRFQIPYGKNIALVGANGVGKTTLLEAIYHQ---IEGIDCSPKVQMAYYRQLAEDMDVSLLQYLMDE--SESFSRAILNNLGLNEALERSCNVLSGGERTKLSLAVLFSTKANMLILDEPTNFLDIKTLEALEMFMNKYPGIILFTSHDTRFVKVSDKKWELTGQSIHDT-------
1_GCN20_YEAST        -IHIDTFDLYVGQRILSNAQLTLSFGHRYGLVGQNGIGKSTLLRALSRR-----ELNVPKHVSILHVEQELLDNREDLDNHLIQISDAEARAASILYGLGFSTEAQQPTNSFSGGWRMRLSLARALFCQPDLLLLDEPSNMLDVPSIAYLAEYLKTYPNTVLTVSHDRAFLNVATDIIYQHNERLDYRGQDF---
2_GCN20_YEAST        IIQLQDVSFGYDNLLLKDVNLDVQMDSRIALVGANGCGKTTLLKIMMEQLRPLKGFVSRNPLRIGYFTQHHSMDTTSAVDWMSKSFPTDEEYRRHLGSFGITGTLLQKMQLLSGGQKSRVAFAALCLNNPHILVLDEPSNHLDTTGLDALVEALKNFNGGVLMVSHDISVIDVCKEIWVSEQGTVKREGTIYDYR
1_EF3A_YEAST_2iwh    DLCNCEFSLAYGKILLNKTQLRLKRARRYGICGPNGCGKSTLMRAIANGQ----VDGFPTQCRTVYVEHDIGTHDTSVLDFVFES--TKEAIKDKLIEFGFTDEMAMPISALSGGWKMKLALARAVLRNADILLLDEPTNHLDTVNVAWLVNYLNTCGITSITISHDSVFLDVCEYIINYEGLKLRKKGNFTEFV
2_EF3A_YEAST         -VKVTNMEFQYPKPQITDINFQCSLSSRIAVIGPNGAGKSTLINVLTGELLPTSGEVYTHECRIAYIKQHAESHDKTPSEYIQWRFQTRKEIEEHCSMLGLDPEIHSRIRGLSGGQKVKLVLAAGTWQRPHLIVLDEPTNYLDRDSLGALSKALKEFEGGVIIITHSAEFTKLTEEVWAVKDGRMTPSGHNWVSG
1_ChvD_254780273     IYHMAGLSKVFGKKVLDDINLSFYPDAKIGILGPNGAGKSTILRIMAGIDKEYNGEAWLATFTVGYLPQEPQLDSKTVKENIIEGVALETEVQVAIESLHCPADGLN-ITSLSGGEKRRVALCKLLLSSPDLLLLDEPTNHLDAETIAWMEKYLREYSGAVLMVTHDRYFLDVTNWILEVDRGKGIPQGNYSAYL
2_ChvD_254780273     VIEAKGISKSYNRLLIDNLSFKLPPGGIVGVIGPNGAGKTTLFRMLTGDESPDSGTIHVGEVCLSYVDQSRSLEEKTVWEDISGGDDHEINSRAYCSAFNFKGGDQQRIGSLSGGQRGRVHLAKILKSGGNVILLDEPTNDLDTETLSALEDALENFAGCAVIISHDRMFLDLATHILAFENGHVEWEGNFEEYE
1_Q8U559_AGRT5       IYHMAGLNKAYGKKVLENIHLSFYPEAKIGILGPNGAGKSTVLRIMAGLDTEYTGEAWVAEARVGYLAQEPQLDQKTVLENVMEGVALDSQVEMAMEALRCPPGDAE-VTNLSGGEKRRVALCKLLLSQPELLLLDEPTNHLDAETIAWLEKHLREYPGAVLMVTHDRYFLDVTGWILELDRGRGIPEGNYSAYL
2_Q8U559_AGRT5       -IEAENISKGYDQLLIEGLSFKLPPGGIVGVIGPNGAGKTTLFRMITGQEQPDDGSIRIGDVQLAYVDQSRALDNKTVWEEISGGNDHEVNSRAYCSTFNFKGGDQQKVGTLSGGQRNRVHLAKMLKSGGNVVLLDEPTNDLDTETLAALEDALENFAGCAVIISHDRMFLDLATHILAFEDSHVEWEGNFEDYE
1_Uup_254781123      ILRLDHISATIGIDLLQDVCLSIKPKERICLVGCNGSGKSTLLKIAAGITEPQSGNVFLHSSRLGYLEQNPDLSFSTISQYIDDTIKAPYSPYSLLKKFNLREQD-R-IENLSVGQTRCVALMKMLISRPDILILDEPTNHLDFRTIHWMEQELLKINSALIFVSHDRRFLELSTTTVWLDRGCLHHDQGFAYFE
2_Uup_254781123      VLEADKITKQYDRLVVKDFSLRIHYGECIGIVGPNGAGKTTLLKLLTGKIKPDCGFITLGTLKIATIDQKRDIDDKSLASYLTGSSGESRHVAGYIKDFLFHPDQHSLMKHLSGGEKMRAIVARVLAQPFNFLIMDEPTNDLDFETLDFLERTITQLQGTILIVSHDRDFLDTVTSTIAAQNGYWIKAGGYSDML
1_Uup_16128916       LISMHGAWLSFSAPLLDNAELHIEDNERVCLVGRNGAGKSTLMKILNREQGLDDGRIIYEQLIVARLQQDPPRNEGSVYDFVAEGIELENRINEVLAQLGLDPNV-A-LSSLSGGWLRKAALGRALVSNPRVLLLDEPTNHLDIETIDWLEGFLKTFNGTIIFISHDRSFIR-NMAIVDLDRGKLVTPGNYDQYL
2_Uup_16128916       VFEMEDVCYQVNKQLVKDFSAQVLRGDKIALIGPNGCGKTTLLKLMLGQLQADSGRIHVGTLEVAYFDQHRELDDKTVMDNLAEGKQKPRHVLGYLQDFLFHPKRMTPVRALSGGERNRLLLARLFLKPSNLLILDEPTNDLDVETLELLEELIDSYQGTVLLVSHDRQFVDTVTECWIFEGGKIGRVGGYHDAR
```

FIGURE S9
